# Data-Driven Recommendation of Optimal Tuning Scheme for Range-Separated Hybrid Functionals in Solution-Phase UV/Vis Absorption Energy Prediction

**DOI:** 10.1021/acs.jctc.5c01044

**Published:** 2025-10-23

**Authors:** Fangning Ren, Pinyuan Li, Xu Chen, Lechen Dong, Fang Liu

**Affiliations:** Department of Chemistry, 1371 Emory University, Atlanta, Georgia 30322, United States

## Abstract

Time-dependent density functional theory (TDDFT) combined with range-separated hybrid (RSH) functionals and a tuned range-separation parameter γ offers a computationally economical approach for high-throughput excited-state property predictions. The γ-tuning procedure in the gas phase is well established. However, no agreement on the best γ-tuning procedure has been made when considering the solvent effect with implicit solvent models like the polarizable continuum model (PCM). To answer that question, this study created a diverse dataset with 937 molecules with experimental solution-phase UV/vis absorption spectra. Three γ-tuning methods, the gas-phase γ-tuning (GPγT), the partial vertical γ-tuning (PVγT), and the strict vertical γ-tuning (SVγT), were evaluated for the ωPBEh functional over the entire dataset. Additional benchmarks are done for the optimally tuned screened range-separated hybrid combined with the PCM approach (SRSH-PCM) and the solvation-mediated tuning procedure (sol-med-OT). Our findings revealed that the optimal γ-values obtained by the PVγT and the SVγT are significantly smaller than the GPγT. This trend holds consistently across all molecules in our dataset, and we explained the origin of this phenomenon. TDDFT calculations with PVγT- and SVγT-tuned γ-values and default global Fock exchange fraction achieve superior performance compared to those using GPγT-tuned or default γ and slightly outperform SRSH-PCM and sol-med-OT with similar or lesser computational cost. Furthermore, we found that the smaller γ-values from SVγT captured the expected 1/(ε*R*) asymptotic behavior in the solution phase, resulting in accurate prediction of solution-phase CT excitations, consistent with the screened asymptote behavior encoded in SRSH-PCM. These results show that SVγT is the best scheme for high-throughput UV/vis absorption spectrum calculations using the ωPBEh functional from a data-driven perspective.

## Graphical abstract

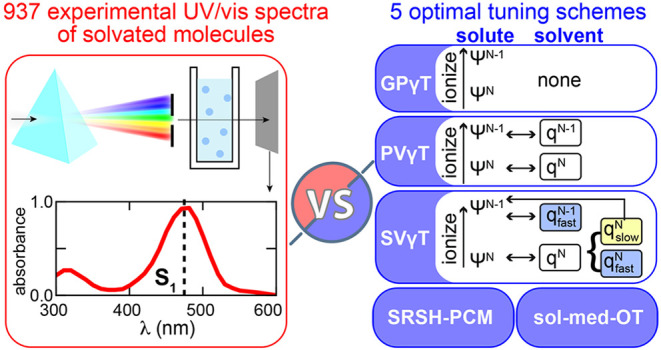

## 1 Introduction

Studying organic molecules’ electronic excited state properties is crucial for many chemical applications: photocatalysts, photosensitizers, photodynamic therapy, and chemical dyes.^1–5^ Fast and accurate computational prediction of the excited state molecular properties in the solution phase allows high-throughput screening and machine-learning (ML) dataset generation, accelerating the discovery of functional molecules in these fields.^1–5^ While high-throughput screening has notably excelled in the ground-state properties for both inorganic^6,7^ and organic chemical systems,^8–12^ reliable large-scale calculation of excited-state properties still faces significant challenges of computational cost and accuracy due to the complex nature of excited states.^13–15^ Similarly, for the purpose of generating training sets for ML, numerous ground-state datasets^16–19^ were curated, whereas fewer excited-state datasets^20,21^ are available.

A practical and cost-effective strategy for high-throughput computation of excited-state properties in large-scale molecular datasets is to employ time-dependent density functional theory (TDDFT)^22–24^ under the Kohn–Sham density functional theory (DFT) framework.^25^ However, TDDFT’s accuracy is highly sensitive to the choice of the exchange-correlation functional.^26–28^ Most general gradient approximation (GGA) functionals and hybrid GGA functionals suffer from varying degrees of delocalization error (DE), which is manifest in deviation from the piecewise linear fractional charge behavior predicted by Janak’s theorem, leading to the erroneous estimation of fundamental and optical gaps.^29,30^ In addition, their incorrect asymptotic behavior leads to severe underestimation of ionization potential (IP) and charge-transfer (CT) excitation energy.^31–33^ A common solution involves the use of range-separated hybrid (RSH) functionals,^34–36^ which enforce the correct asymptotic behavior using an Ewald-style partition:

1 $$\frac{1}{r_{12}}=\frac{1-\left[\alpha +\beta \mathrm{erf}\left(\gamma r_{12}\right)\right]}{r_{12}}+\frac{\alpha +\beta \mathrm{erf}\left(\gamma r_{12}\right)}{r_{12}}$$

Here, the *r*_12_ represents the two-electron Coulomb operator. α, β, and γ are parameters. The first term controls the short-range (SR) interaction computed by GGA or hybrid GGA functionals. This term decays to 1 – (α + β) as *r*_12_ approaches infinity. The second term controls the long-range (LR) interaction that is governed by Hartree–Fock exchange, which starts from α and smoothly increases to α + β as *r*_12_ approaches infinity. Accordingly, α and α + β stand for the fractions of Fock exchanges at SR and LR. γ is the range-separation parameter, with units of inverse length, and 1/γ defines the characteristic length scale of the short-range interaction. In some other context, the range-separation parameter is also denoted by ω or μ. This partition guarantees that hybrid functionals exhibit the correct long-range asymptotic behavior and improve their accuracy for CT excitation energies.^37–39^ Each DFT functional typically possesses its own default set of α, β, and γ, determined by calibrating against some benchmark datasets.

However, the accuracy of RSH functionals on individual systems depends on α, β, and γ. When using the default α and β, the optimal γ that minimizes the DE is system-dependent,^40,41^ and using the default γ in RSH functionals usually results in inaccurate non-CT excited state energies, even worse than hybrid GGA functionals.^26–28,42^ To overcome this limitation, the γ-tuning procedure was developed.^43^ This procedure aims to find an optimum γ-value for each system that enforces Koopmans’ theorem by minimizing the following objective function *J* ^2^(γ):

2 $$J^{2}\left(\gamma \right)={\left({\text{ϵ}}_{\mathrm{HOMO}\left(N\right)}^{\gamma }+\mathrm{IP}\left(N;\gamma \right)\right)}^{2}+{\left({\text{ϵ}}_{\mathrm{HOMO}\left(N+1\right)}^{\gamma }+\mathrm{IP}\left(N+1;\gamma \right)\right)}^{2}$$

3 IP(N;γ)=E(N−1;γ)−E(N;γ)

Here, ϵ_HOMO(*N*)_^γ^ is the highest occupied orbital (HOMO) energy of an N-electron system, IP­(*N*; γ) is the ionization potential (IP) of this neutral N-electron system, and *E*(*N*; γ) is the total electronic energy. The corresponding quantities with *N* + 1 and *N* – 1 notations in the brackets are for the anionic (*N* + 1 electrons) and cationic (*N* – 1 electrons) systems, respectively. In terms of DE, γ-tuning reinstates the piecewise linear fractional charge behavior predicted by Janak’s theorem, thereby minimizing the DE.^29^ This scheme enables DFT to achieve accuracy comparable with high-level wave function-based methods,^41,43,44^ thus enabling accurate high-throughput calculations at a reasonable cost. Note that “γ-tuning” is synonymous with “ω-tuning” or “μ-tuning” in other articles, where the range-separation parameter is denoted by ω or μ. In the following, we will consistently use “γ-tuning” to refer to this procedure.

Despite its success, the γ tuning procedure is typically conducted in vacuum, which does not reflect real experimental scenarios. In most experimental settings, the target molecular system is studied in solution, where the solvent effects on the solute’s excited state properties, e.g., UV/vis spectrum, are often not negligible.^45–47^ Explicitly considering these solvents significantly increases the computational cost for both DFT and TDDFT calculations. Consequently, implicit solvent models, such as the polarizable continuum model (PCM), are commonly employed as practical alternatives.^48–50^ To account for environmental effects, the γ-tuning procedure should be integrated with the PCM.^51^ Various methodologies have been developed to perform γ-tuning for molecules in the solution phase.^51–55^ However, no consensus has been reached regarding the optimal tuning approach. These methodologies primarily differ in how they adapt the quantities in eqs 2 and 3 to the solvent environment.

As illustrated in Figure 1, the most straightforward approach is to utilize the optimal γ value obtained in the gas phase for solution-phase TDDFT calculations, a method termed “gas-phase γ-tuning (GPγT)”.^56^ While GPγT is conceptually simple, it fails to accurately reproduce the experimental optical gap in polar solvents.^57^ Another conceptually straightforward scheme is the partial vertical γ-tuning (PVγT), where all quantities in eqs 2 and 3 are calculated in the presence of equilibrium PCM with the static dielectric constant ε. In the implicit-solvent picture of Figure 1, this means that upon the solute’s ionization from *N* to *N* – 1 electron state, the polarization charges caused by the solvent’s electron polarization *q*_fast_^*N*^ and conformation relaxation *q*_slow_^*N*^ relax to those of the charged state (*q*_fast_^*N–*1^, *q*_slow_^*N–*1^). Because the solvent nuclear coordinates are allowed to relax on the joint solute–solvent potential-energy surface, the ionization is not strictly vertical, hence, “partial vertical”. PVγT has been scrutinized due to the very small γ value, which is suspected to reintroduce the DE associated with pure-GGA functionals.^51,55,58,59^ The strict vertical γ-tuning (SVγT) applies nonequilibrium PCM correction for a more realistic consideration of the ionization (electron addition) process.^55,60,61^ In SVγT, the molecule’s neutral state energies [ ${\text{ϵ}}_{\mathrm{HOMO}\left(N\right)}^{\gamma }$ and *E*(*N*; γ)] are calculated with equilibrium PCM using static dielectric constant ε. SVγT treats the neutral state with equilibrium PCM (ε) to obtain ${\text{ϵ}}_{\mathrm{HOMO}\left(N\right)}^{\gamma }$ and *E*(*N*; γ). Then it evaluates the cation (anion) state with nonequilibrium PCM using the optical dielectric ε_∞_, updating only $q_{\mathrm{fast}}^{N}$ to $q_{\mathrm{fast}}^{N-1}$ $\left(q_{\mathrm{fast}}^{N+1}\right)$ while keeping $q_{\mathrm{slow}}^{N}$ unchanged, and yielding the energetics of the *N* + 1 and *N* – 1 state [ϵ_HOMO(*N*+1)_^γ^, *E*(*N* + 1; γ), and *E*(*N* – 1; γ)]. With both the solute geometry and the solvent nuclear configuration fixed during ionization, this procedure is strictly vertical and mirrors the Franck–Condon picture of UV/vis excitation. Therefore, SVγT in principle should yield a more accurate description of the spectrum. However, benchmark studies by Sachse et al.^60^ based on representative organic photovoltaic molecules report that PVγT and SVγT often yield very similar UV/vis spectra, with only PVγT capturing the gap-renormalization effect. The putative theoretical superiority of SVγT therefore still needs to be assessed on larger and more diverse datasets.

**Figure 1:**
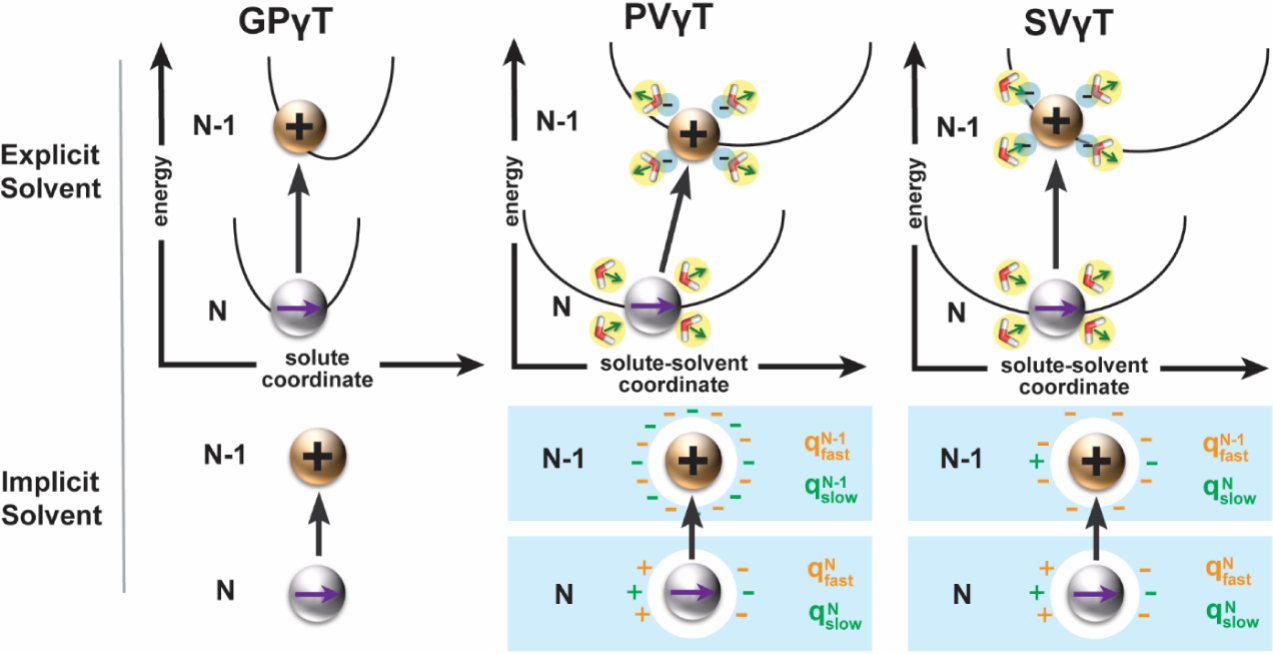
Schematic of three γ-tuning schemes under explicit and implicit solvation. Top row: potential-energy sketches for the ionization process from the neutral state (N electron) to cation state (*N* – 1 electron) as “vertical” processes. Bottom row: the corresponding treatments in the implicit solvent model, such as PCM. Orange and green charges are fast and slow polarization charges induced by the N-electron solute, denoted as $q_{\mathrm{fast}}^{N}$ and $q_{\mathrm{slow}}^{N}$ , respectively. In an implicit model, $q_{\mathrm{fast}}^{N}$ $\left(q_{\mathrm{slow}}^{N}\right)$ represents the polarization charges generated from the solvent’s electronic (nuclear) degree of freedom with the solvent under the N-electron state. GPγT (left): tuning in the gas phase with no solvent response; PVγT (middle): both solvent electrons and nuclei fully relax upon ionization, with both $q_{\mathrm{fast}}^{N}$ and $q_{\mathrm{slow}}^{N}$ equilibrate to the cationic state and change to $q_{\mathrm{fast}}^{N-1}$ and $q_{\mathrm{slow}}^{N-1}$ , respectively. SVγT (right): only the solvent’s electron responds to the ionization, with only $q_{\mathrm{fast}}^{N}$ changes to $q_{\mathrm{fast}}^{N-1}$ while $q_{\mathrm{slow}}^{N}$ remains constant.

A different approach for incorporating the solvent effect is the optimally tuned screened RSH combined with PCM­(SRSH-PCM).^62^ In SRSH-PCM, the optimal γ value for a given system is obtained through GPγT without changing α or β. When performing the solution-phase TDDFT calculation, after enabling the PCM, the LR Fock exchange α + β is set to 1/ε to mimic the dielectric screening effect of the environment. It is also possible to tune the α and γ simultaneously to find the optimal α, γ pair, represented by the solvation-mediated tuning procedure (sol-med-OT) proposed by Joo et al.^63^ and the OT-SRSH-PCM approach proposed by Bhandari et al.^64^ These methods avoid the suspiciously small γ value and have been successful in predicting the gap-renormalization effect in solid-state calculations,^65^ but their performance in the solution phase has yet to be thoroughly evaluated.

Although all schemes have demonstrated some advantages, it is still difficult to conclude the most effective γ-tuning scheme. This is because the benchmark studies of different γ-tuning schemes in predicting excited-state properties have been conducted only on small sets of molecules or individual systems.^61,66,67^ Their performance relative to experimental results has not been examined for large, diverse datasets. Consequently, the optimal γ-tuning scheme within PCM for data-driven molecular discovery applications, such as large-scale, high-throughput screening or generating datasets for training machine learning models, remains uncertain.

This study aims to address these gaps using a data-driven approach. We curate a diverse dataset of 937 molecules with their solution-phase experimentally measured UV/vis absorption spectra. We apply three different γ-tuning schemes (GPγT, PVγT, SVγT) to the RSH functional ωPBEh on these molecules and determine their optimal γ parameters under each tuning scheme. Absorption energies are computed via TDDFT with linear-response PCM^49^ (LR-PCM) at nonequilibrium solvation^68,69^ and compared with the experimental spectrum. This study finds that both PVγT and SVγT yield much smaller optimal γ than GPγT, with most of the optimal γ from PVγT being close to zero. Comparing the results of TDDFT with the experimental data, PVγT and SVγT exhibit very similar accuracy, which is markedly improved relative to that of GPγT and the default γ. Additionally, it is also found that small γ values from SVγT can reproduce the expected 1/(ε*R*) asymptotic behavior in the solution phase, facilitating the prediction of CT excitations in solution. These results underscore the advantage of the SVγT in solution-phase absorption energy prediction for neutral organic molecules.

## 2 Method

### 2.1 Dataset Curation

The experimental UV/vis data were extracted from a database compiled by Bread et al.^70^ This dataset originally includes 8487 records extracted from 402,034 published research articles with the text-mining toolkit ChemDataExtractor.^71^ Here, we postprocessed their dataset as follows: aiming at solution-phase γ tuning, we first removed entries without solvent information, retaining 1446 records out of 8487 records. Then, we removed very small solutes containing less than 5 heavy atoms, because these substances usually correspond to solvents, salts, and small molecule reactants that are not typical photoactive molecules in chemical applications. Also, due to the algorithm used in ChemDataExtractor, SMILES strings containing fewer than 5 heavy atoms are prone to data entry error and must be discarded to ensure dataset quality. Although some ionic solutes are also of chemical interest, they experience significantly stronger solvent effects than neutral molecules, which implicit models fail to capture accurately.^72^ Since ionic solutes constitute only 8.77% of the dataset, we chose not to consider them in this study to ensure the consistency of our conclusions over neutral molecules. Finally, we filtered metal-containing solutes, such as transition metal complexes, because an accurate description of their optical property usually requires multireference methods,^73^ and TDDFT is expected to result in large errors due to its single-reference nature. For the remaining 997 entries, we conducted a very careful manual screening and ultimately discarded 60 erroneous entries and corrected 387 absorption wavelengths, resulting in a refined dataset consisting of 937 distinct solutes in 9 different solvents. Our dataset is focused on medium to large neutral organic molecules, which covers a wide range of systems of interest, such as fluorescent dyes,^74^ optoelectronic materials,^75^ and organic photoredox catalysts.^76^

Our refined dataset contains 937 distinct solutes solvated in 9 different solvents with varying dielectric constants ranging from 2.02 to 46.71. Solvents in our dataset with a dielectric constant less than 20 are denoted as nonpolar solvents, including cyclohexane, toluene, chloroform, tetrahydrofuran (THF), and dichloromethane (DCM). Those with a dielectric constant greater than 20 are considered polar solvents, such as ethanol, methanol, *N*,*N*-dimethylformamide (DMF), and dimethyl sulfoxide (DMSO). As illustrated in Figure 2, the dataset is divided into two subsets based on solvent polarities. The nonpolar solvent subset encompasses solute sizes ranging from 12 heavy atoms (M259) to 96 heavy atoms (M40), while the polar solvent subset contains solutes varying from 11 atoms (M834) to 116 atoms (the largest). Notably, both subsets demonstrate similar solute size distributions, with the most frequently observed size being about 25 heavy atoms. For each system, only the first visible excitation peak of the experimental spectrum, denoted as Δ*E*_peak_, is compared with the corresponding calculation. The Δ*E*_peak_ of both subsets also exhibit similar distributions, spanning from 1.42 eV (M1400) to 5.08 eV (M443) in the nonpolar subset and from 1.38 eV (M60) to 4.96 eV (M976) in the polar subset.

**Figure 2:**
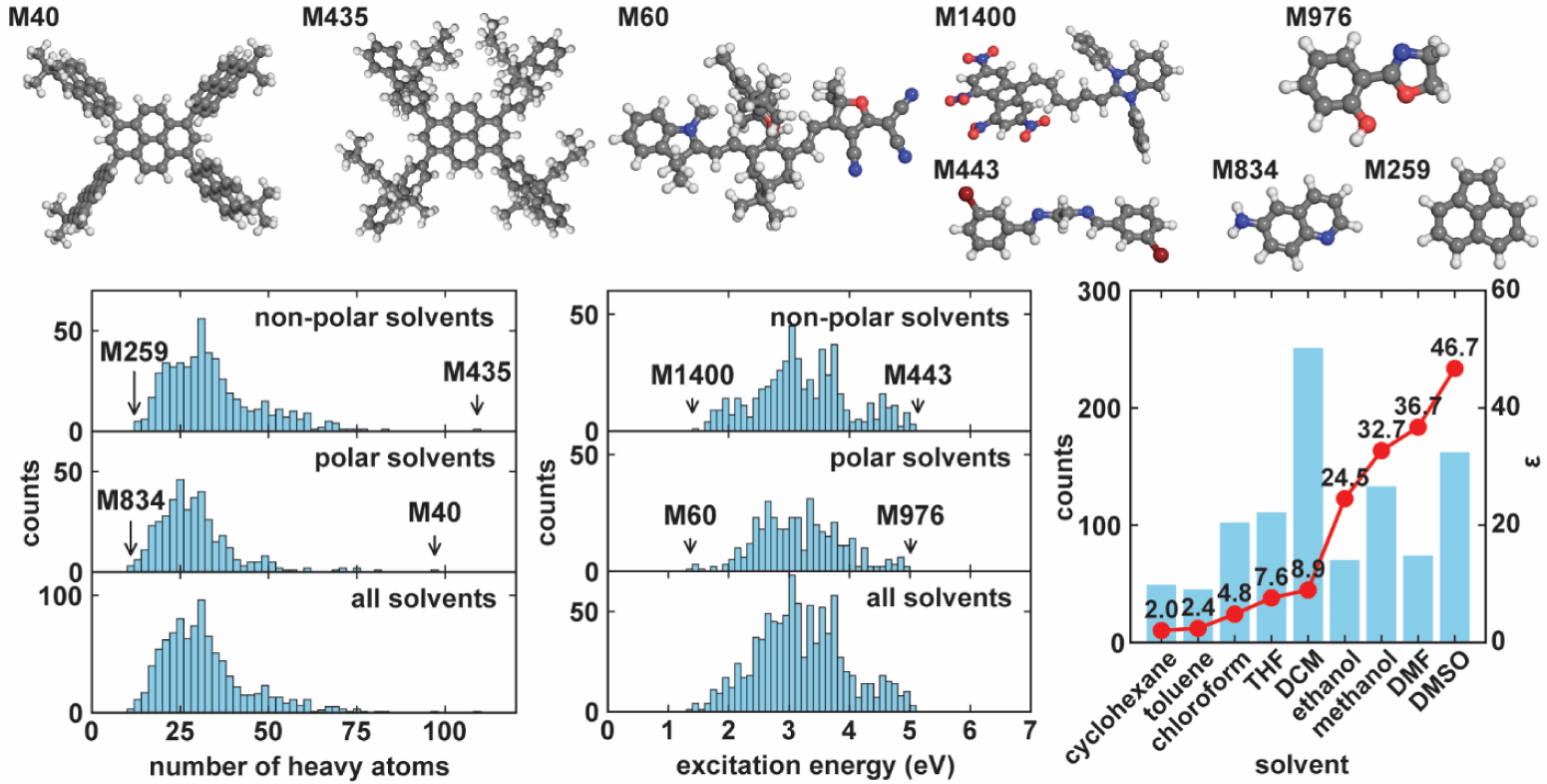
Properties of solvated molecules in the refined dataset. (Top) The 3D structures of representative molecules from our dataset. Atoms were color-coded based on their element identity: C in gray, H in light gray, O in red, S in yellow, N in blue, and Br in dark red. These molecules have been chosen to represent both ends of the spectrum in terms of heavy atom count and Δ*E*(S_1_). (Bottom left) The bar plots map out the distribution of the number of heavy atoms of solutes in our dataset in the polar and nonpolar solvent subsets. (Bottom middle) The bar plots display the distribution of Δ*E*(S_1_) in the whole dataset and within the polar and nonpolar subsets. (Bottom right) The bar plot shows the frequency count of solvents in our dataset, with their dielectric constants, ε, labeled and plotted on a red curve.

### 2.2 DFT Calculations

All geometry optimizations and ground-state energy calculations for the neutral, cationic, and ionic species were performed at the DFT level of theory with the RSH functional ωPBEh,^39^ as the QC. The default α, β, and γ values for ωPBEh are 0.2, 0.8, and 0.2 a_0_^–1^, respectively, where a_0_^–1^ denotes the inverse of the atomic length unit Bohr. Default ωPBEh ensures the LR Fock exchange fraction α + β to be 1, which may not hold in other RSH functionals such as CAM-B3LYP.^54^ The 6-31G* basis function^77–79^ was used for all elements except for Br and I, which were treated with the LANL2DZ effective core potential. The geometry optimizations were performed with the conductor-like polarizable continuum model (C-PCM) with the improved Switching-Gaussian formalism (ISWIG),^80^ implemented on GPUs.^81,82^ with corresponding static dielectric constants for the solvents. All geometry optimizations utilized ωPBEh with default α, β, and γ values and the same basis set. The solute cavities for C-PCM were built based on the default radii values by Bondi for nonmetals^83^ and standard van der Waal radii for metals collected from a published database,^84^ multiplied by a scaling factor of 1.2. Depending on the γ-tuning schemes, DFT calculations were performed in the gas phase, with equilibrium PCM, or with nonequilibrium PCM. In these calculations, each solvent’s static dielectric constant, ε, was directly taken from the E_T_(30) dataset,^85^ whereas the optical (fast) dielectric constant, ε_∞_ was calculated as the square of its refraction index, *n*, of the E_T_(30) dataset.^85^ Detailed procedure of γ-tuning will be explained in the following section. All quantum chemistry calculations were performed with the GPU-accelerated quantum chemistry package, TeraChem.^86^ UV/vis excitation energy calculations were performed with TDDFT with the Tamm–Dancoff approximation (TDA)^87^ using the same functional and basis set as the ground state DFT calculations. TDA was used because it is known to improve the accuracy of predicted vertical excitation energies compared with full TDDFT with lower computation costs,^88^ and we also repeated the TDDFT calculation of SVγT with full TDDFT enabled to quantitatively assess the impact of TDA. Unless otherwise stated, all TDDFT calculations were performed using TDA. Nonequilibrium linear-response PCM^68,69^ is used in all TDDFT calculations with the optical dielectric constant, ε_∞_. For each TDDFT calculation, the first 10 excited states with their energy and oscillator strengths were calculated. The corresponding absorption spectrum was plotted by convoluting the excitations using a Gaussian function with a full width at half-maximum of 0.20 eV. The height of the Gaussian functions was set proportional to the corresponding state’s oscillator strength. As most absorption spectra reported in the literature have no significant vibronic structures, we neglected vibronic contributions as their inclusion would require computationally demanding evaluation of the Hessian matrix.

Assigning the experimentally observed peaks to the corresponding bright transitions for the entire dataset requires chemical intuition and tremendous human labor, and some short-wavelength experimental absorption peaks may not correspond to the first 10 excited states obtained from our calculations. Moreover, some observed absorption peaks may be contributed by multiple nearly degenerate bright states. Therefore, to make a reasonable comparison between computation and experiment, we only compare the first visible absorption peak (Δ*E*_peak_) between the calculated and experimental spectra, as it is closest to the optical gap of the molecule that people are usually interested in. It is worth noting that if the S_1_ is a dark state, it does not affect the spectrum. Therefore, the resulting Δ*E*_peak_ does not necessarily correspond to the S_0_–S_1_ transition energy, but could instead be associated with S_2_, S_3_, or even higher excited states. For each system, we evaluated the excited state that has the largest contribution to the first observed absorption peak in the simulated spectrum after Gaussian convolution (Figure S1). Among all 937 entries, approximately 60% of Δ*E*_peak_ values originate from S_1_, 24% from S_2_, and the remainder from S_3_ or higher excited states. These proportions are consistent across all four γ-tuning schemes, default γ, GPγT, PVγT, and SVγT, indicating that the identity of the contributing state is largely unaffected by the choice of tuning procedure. A comparison between the simulated spectrum (including Gaussian functions for S_1_–S_10_) and the experimental spectrum of M472 is included in Figure S2, where the experimental absorption peak at 345 nm is associated with the S_3_ state calculated by SVγT.

### 2.3 γ-Tuning Procedure

The geometries of solutes were first optimized at the ground state with equilibrium PCM using the default γ = 0.2 a_0_^–1^ before the γ-tuning, with the D3 dispersion correction used to account for the dispersion interaction.^89^ Then, for each tuning scheme, the optimal γ was searched to minimize the loss function *J* ^2^(γ) according to eq 2 at fixed geometry. We first computed *J* ^2^(γ) for γ from 0.00 a_0_^–1^ and 0.60 a_0_^–1^ with a step size of 0.15 a_0_^–1^ and choose the γ with the smallest loss. In the second round, we computed the loss for γ within a ± 0.15 a_0_^–1^ range of the γ obtained in the first round with a smaller step size of 0.02 a_0_^–1^. The γ that yields the smallest loss in the second round was set to be the optimum γ-value.

The loss function *J* ^2^(γ) requires the IP and the HOMO energies of the solute, whose calculation protocol depends on the tuning schemes illustrated in Figure 1. For the gas-phase tuning (GPγT), we performed the ground-state calculations for the molecule’s neutral (*N* electrons), anionic (*N* + 1 electrons), and cationic (*N* – 1 electrons) states at each γ in the gas phase. Then, we extracted the HOMO energies $[{\text{ϵ}}_{\mathrm{HOMO}\left(N\right)}^{\gamma }$ and ${\text{ϵ}}_{\mathrm{HOMO}\left(N+1\right)}^{\gamma }]$ and the IPs [IP­(*N*; γ) and IP­(*N* + 1; γ)] for the neutral and anionic states and inserted them in eq 3.

In the PVγT and the SVγT, C-PCM was used to model the solvent effect in all QM calculations. The PVγT used the equilibrium PCM with the static dielectric constant ε to construct the solvent reaction field for neutral, cation, and anion states, where both the nuclear and electron degrees of freedom were allowed to respond. In contrast, the SVγT used equilibrium PCM to calculate the molecule’s neutral state only and stored the corresponding solvent polarization charges. Then, the solvent polarization charges were partitioned into the fast (electronic) and slow (nuclear) components, *q*_fast_^(*N*)^ and *q*_slow_^(*N*)^.^90^ When performing the DFT calculations of the cationic and anionic states, the dielectric constant of C-PCM was set to the solvent’s optical dielectric constant ε_∞_, with the solvent electric field induced by *q*_slow_^(*N*)^ loaded as a fixed external field. This approach allowed only the solvent’s electronic degree of freedom to respond to the solute’s electron addition or removal process.

### 2.4 Evaluating the One-Particle Picture

As discussed by Presselt et al.,^60^ one of the goals for γ-tuning is to reinstate the so-called “one-particle picture”, which presumes that the removal of an electron from the HOMO or the addition of an electron to the LUMO will not influence other molecular orbitals.^41^ Therefore, the HOMO–LUMO excitation does not affect other orbitals as well. Hence, substantial deviations from the one-particle picture indicate that the γ-tuning is not physically meaningful, even if it yields results that agree with the experiment. Therefore, one needs to analyze the validity of γ-tuning by testing the one-particle picture, i.e., whether

$$\rho^{N}\left(\mathrm{r⃗}\right)-\rho^{N-1}\left(\mathrm{r⃗}\right)\equiv \Delta \rho \left(\mathrm{r⃗}\right)={|\phi_{\mathrm{HOMO}}^{N}\left(\overset{⇀}{r}\right)|}^{2}$$

holds. Here, *ρ^N^* (*r⃗*) and *ρ* ^*N*–1^(*r⃗*) is the total electron density of the same molecule’s neutral and cationic states, respectively. ${|\phi_{\mathrm{HOMO}}^{N}\left(\overset{⇀}{r}\right)|}^{2}$ is the square of the HOMO for the N-electron neutral state, which is expected to be identical to the density difference after ionization.

We aimed at testing whether the γ-tuning is physically meaningful for molecules in our dataset, i.e., whether the one-particle picture holds for these molecules in the solution phase with PCM enabled. Considering that our TDDFT calculations used nonequilibrium PCM to mimic the physical picture of UV/vis absorption, the ground-state nonequilibrium PCM was used to calculate *ρ* ^*N*–1^(*r⃗*), i.e., only *q*_fast_^(*N–1*)^ responds to the ionization, while retaining the slow component of the solvent polarization change induced by the neutral molecule, *q*_slow_^(*N*)^. Inspired by the density-based comparison method proposed by Presselt et al.,^60^ we compared Δ*ρ*(*r⃗*) and ${|\phi_{\mathrm{HOMO}}^{N}\left(\overset{⇀}{r}\right)|}^{2}$ based on their atomic Hirshfeld population.^91^ The Hirshfeld population partitions the real space to each atom based on the atom’s promolecular electron density, which reflects the distribution of Δ*ρ*(*r⃗*) and ${|\phi_{\mathrm{HOMO}}^{N}\left(\overset{⇀}{r}\right)|}^{2}$ over the molecule. Compared to wave function-based population analysis, such as Mulliken population analysis,^92^ the pure-density-based Hirshfeld method provides a more direct representation of electron density and exhibits lower sensitivity to basis functions. Numerically, the Hirshfeld population analysis of Δ*ρ*(*r⃗*) and ${|\phi_{\mathrm{HOMO}}^{N}\left(\overset{⇀}{r}\right)|}^{2}$ were all evaluated using Multiwfn^93^ with its built-in sphericalized atomic densities in free-states. The real-space electron density was integrated with Becke’s multicenter numerical integration algorithm^94^ using a total of 32 550 spherical grids per atom (75 radial grids and 434 angular grids), with a radius cutoff 10 a_0_^–1^ to save time during the numerical integration. Because the integration algorithm assigns a spherical grid set with identical radial and angular grid density to the center or each atom, the total number of integration points for a molecule scales proportionally with its number of atoms, while the per-atom grid density is kept constant to ensure uniform numerical accuracy across all systems. The squared Pearson correlation coefficient (*R* ^2^ between the atomic Hirshfeld population of Δ*ρ*(*r⃗*) and ${|\phi_{\mathrm{HOMO}}^{N}\left(\overset{⇀}{r}\right)|}^{2}$ were then calculated for each system as a quantitative measure of consistency with the one-particle picture. γ-tuning results with *R* ^2^ > 0.90 are considered as physically meaningful from the perspective of the one-particle picture.

### 2.5 Asymptote Behavior Evaluation

To systematically evaluate the CT state asymptote behavior of DFT functionals, additional DFT calculations were performed on a set of ethylene–tetrafluoroethylene (ETH–TFE) dimers, one of the most classic systems for studying the CT excitations.^38,95^ Both molecules were first placed in the xOz plane with their geometric center moving to the origin. Then the TFE molecule was moved along the positive *z*-axis for 5 Å to 9 Å with a step of 0.5 Å, creating a total of 9 conformers with different separation distances. TDDFT calculations were performed at the ωPBEh/6-31G* level using different γ values ranging from 0.0 a_0_^–1^ to 0.2 a_0_^–1^. The same calculations were performed in the gas phase, cyclohexane, chloroform, and DMSO, and the solvent effects were simulated using nonequilibrium LR-PCM. For each TDDFT result, the CT state to compare was selected as the first state with the y-component of its unrelaxed excited state dipole moment greater than 12.5 D.

Since our focus in this work is UV/vis absorption spectrum prediction, a fast process with nonequilibrium solvation during the electronic excitation, the reference solution-phase CT excitation energy should be obtained under nonequilibrium solvation conditions. The CT excitation energy with correct asymptotic behavior, denoted as $\Delta E_{\mathrm{CT}}^{\mathrm{asymptotic}}$ , relative distance between the ETH and TFE molecules can be approximated as

4 $$\Delta E_{\mathrm{CT}}^{\mathrm{asymptotic}}\left(R,\varepsilon ,\varepsilon_{\infty }\right)={\mathrm{IP}}_{\mathrm{ETH}}\left(\varepsilon ,\varepsilon_{\infty }\right)+{\mathrm{EA}}_{\mathrm{TFE}}\left(\varepsilon ,\varepsilon_{\infty }\right)-\frac{1}{\varepsilon_{\infty }R}$$

Here, given the solvent’s static dielectric constant *ε* and optical dielectric constant ε_∞_, IP_ETH_(ε,ε_∞_) is the solution-phase ionization potential of the ETH with the cationic form treated with nonequilibrium solvation, and EA_TFE_(ε,ε_∞_) is the solution-phase electron affinity of TFE with the anionic form treated with nonequilibrium solvation. The last term, (1/(ε_∞_ *R*), approximates the Coulombic attraction between the two separated moieties with the dielectric medium. When ε = ε_∞_ = 1, eq 4 falls back to the Mulliken rule of CT excitations in vacuum.^96^ eq 4 assumes that only the electronic degree of freedom of the solvent reacts to the instantaneous CT excitation process, corresponding to the nonequilibrium solvation. Therefore, the last term only contains the optical dielectric constant.

To test whether the TDDFT calculated CT excitation energies with different γ, denoted by $\Delta E_{\mathrm{CT}}^{\mathrm{TDDFT},\gamma }$ , comply with eq 4. We also computed the reference value of IP_ETH_ and EA_TFE_ in eq 4 within different solvents by the Coupled Cluster Singles and Doubles with Triple correction^97^ (CCSD­(T)) with the aug-cc-PVTZ basis set.^98,99^ Ground-state nonequilibrium PCM was used to simulate the solvent’s response to the solute’s electron addition and removal. Since eq 4 approximates the electrostatic interaction of the electron and hole as the interaction of a pair of opposite point charges, it is accurate only when the two molecules are very far apart. As a comparison, we propose another approach to analyze the electron–hole interaction by representing the ETH cation and TFE anion with their atomic charges. Specifically, we fitted their ChElPG^100^ atomic charges based on their CCSD­(T) density. Therefore, a more accurate approximation of CT energies can be obtained compared with eq 5:

5 $$\Delta E_{\mathrm{CT}}^{\mathrm{ChElPG}}\left(R,\varepsilon ,\varepsilon_{\infty }\right)={\mathrm{IP}}_{\mathrm{ETH}}\left(\varepsilon ,\varepsilon_{\infty }\right)+{\mathrm{EA}}_{\mathrm{TFE}}\left(\varepsilon ,\varepsilon_{\infty }\right)+\underset{i\mathrm{in ETH}}{\sum }\underset{j\mathrm{in TFE}}{\sum }\frac{q_{i}^{\mathrm{ChElPG}}q_{j}^{\mathrm{ChElPG}}}{\varepsilon_{\infty }r}$$

Here, *i* and *j* are atom indices in ETH and TFE, respectively, and $q_{i}^{\mathrm{ChElPG}}$ and $q_{j}^{\mathrm{ChElPG}}$ are corresponding ChElPG atomic charges. The summation goes over each atomic pair between ETH and TFE to calculate the Coulombic interaction energy. The CCSD­(T) calculation and ChElPG charge fitting were performed using the Q-Chem 6.0 package.^101^

## 3 Results

### 3.1 The Optimum γ-Value Distribution

The results of GPγT, the PVγT, and the SVγT for the dataset are presented as histograms in Figure 3. The optimum γ-values of GPγT range from 0.10 a_0_^–1^ to 0.22 a_0_^–1^, resembling a normal distribution with the mode at 0.14 a_0_^–1^ to 0.16 a_0_^–1^. The PVγT and SVγT results show much narrower distributions. Similar to previous studies,^59,64,102^ we find that most of the optimum γ-values for PVγT are exactly 0.0 a_0_^–1^, and most of the optimal γ-values for SVγT are below 0.1 a_0_^–1^. Despite the different treatments of the solvent effects, both PVγT and SVγT’s results show the trend that incorporating solvent effects in the optimal tuning process will result in smaller optimum γ-values.

**Figure 3:**
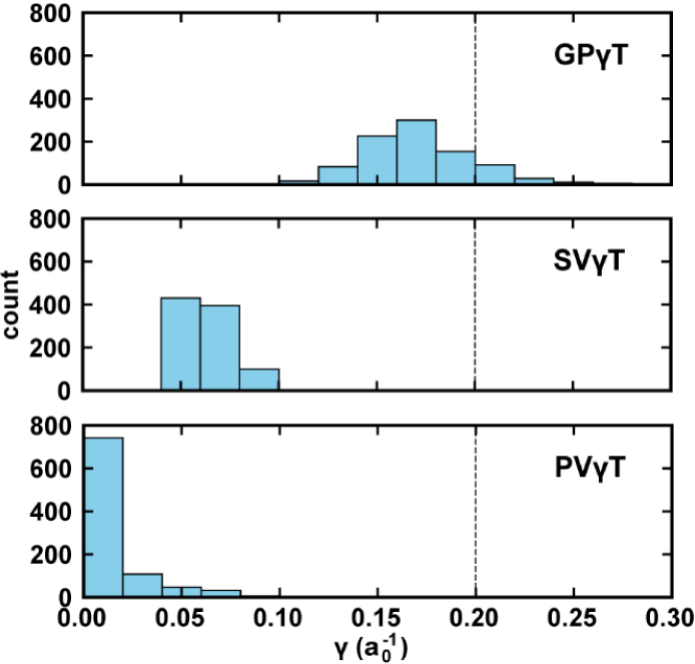
Histogram of the distribution of optimum γ values (in a_0_^–1^) obtained by GPγT, SVγT, and PVγT. A vertical dashed line in each figure indicates the default γ = 0.20 a_0_^–1^.

Our optimal γ values are obtained through minimizing both terms in eq 2 to satisfy Janak’s theorem, which is slightly different than the original IP-tuning procedure that only minimizes the first term to enforce Koopman’s theorem.^38^ Another approach is to replace the second term in eq 3 with ${\left({\text{ϵ}}_{\mathrm{LUMO}\left(N\right)}^{\gamma }+\mathrm{EA}\left(N;\gamma \right)\right)}^{2}$ , where ${\text{ϵ}}_{\mathrm{LUMO}\left(N\right)}^{\gamma }$ and EA­(*N*; γ) are the LUMO energy and electron affinity of the N-electron state, respectively.^41^ We conducted all γ-tuning procedures by only minimizing the first term in eq 2 and found that changing the loss function does not affect the optimal γ by more than 0.02 a_0_^–1^ for all three tuning schemes (Figure S3). Therefore, the form of the loss function has a negligible effect on the optimal γ distribution obtained by the three procedures we evaluated here. The average *J* ^2^ obtained by all three tuning schemes is only 0.005 eV^2^ (Figure S4), indicating the difference between ${\text{ϵ}}_{\mathrm{HOMO}\left(N\right)}^{\gamma }$ and IP­(*N*; γ) is around 0.05 eV for most systems. Therefore, a grid spacing of 0.02 a_0_^–1^ is sufficient to find the optimal γ that reinstates Janak’s theorem. Even for the 49 solutes with the *J* ^2^(γ) from at least one of the 3 schemes exceeds 0.02 eV^2^, our approach can find the global minimum of *J* ^2^(γ) (see Figure S5 for an extreme case: M33 in GPγT). Therefore, our γ-tuning protocol is sufficiently precise for all 937 systems examined in this study.

To understand the influence of solvent effects on the optimum γ-values, we plot the negative HOMO eigenvalues (−ϵ_HOMO_) and the IPs obtained at different γ-values for the neutral (N electrons) and anion (*N* + 1 electrons) states of M117, a typical organic solute in our dataset (Figure 4). The solvent used here is DMF, a polar, nonprotic solvent with ε = 36.7 and ε_∞_ = 2.04. The intersection points between the −ϵ_HOMO_ and the IP curves determine the optimum γ-values defined in eq 3). Across different tuning schemes (different panels in Figure 4), the intersection points shift toward smaller γ to varying degrees due to different approaches to treat solvent effects when evaluating −ϵ_HOMO_ and IP. While the $-{\text{ϵ}}_{\mathrm{HOMO}\left(N\right)}^{\gamma }$ remains almost unchanged, all IP­(*N*; γ) decreases significantly for all γ as the tuning scheme switches from GPγT to SVγT and PVγT, with an increasing level of solvent relaxation considered. This phenomenon has also been observed in some other contexts.^58^ The negligible response of $-{\text{ϵ}}_{\mathrm{HOMO}\left(N\right)}^{\gamma }$ to the PCM field can be explained by the fact that the polarization charge induced by neutral molecules is usually negligible even in a polar solvent with a large ε. Hence, neither the total energy IP­(*N*; γ) nor the HOMO energy ${\text{ϵ}}_{\mathrm{HOMO}\left(N\right)}^{\gamma }$ is significantly impacted by PCM. In contrast, the cationic state (*N*–1 electrons) is better stabilized by the PCM field, resulting in a significant decrease in the total energy *E*(*N* – 1; γ) as a PCM field is applied. Since SVγT uses a smaller ε when calculating the cationic state, the corresponding impact on E(N−1;γ) is less than PVγT. Combined together, IP­(*N*; γ) = *E*(*N* – 1; γ) – *E*(*N*; γ) significantly decreases as the tuning scheme switches from GPγT to SVγT and PVγT, and the intersection of $-{\text{ϵ}}_{\mathrm{HOMO}\left(N\right)}^{\gamma }$ and IP­(*N*; γ) curves move toward a smaller γ.

**Figure 4:**
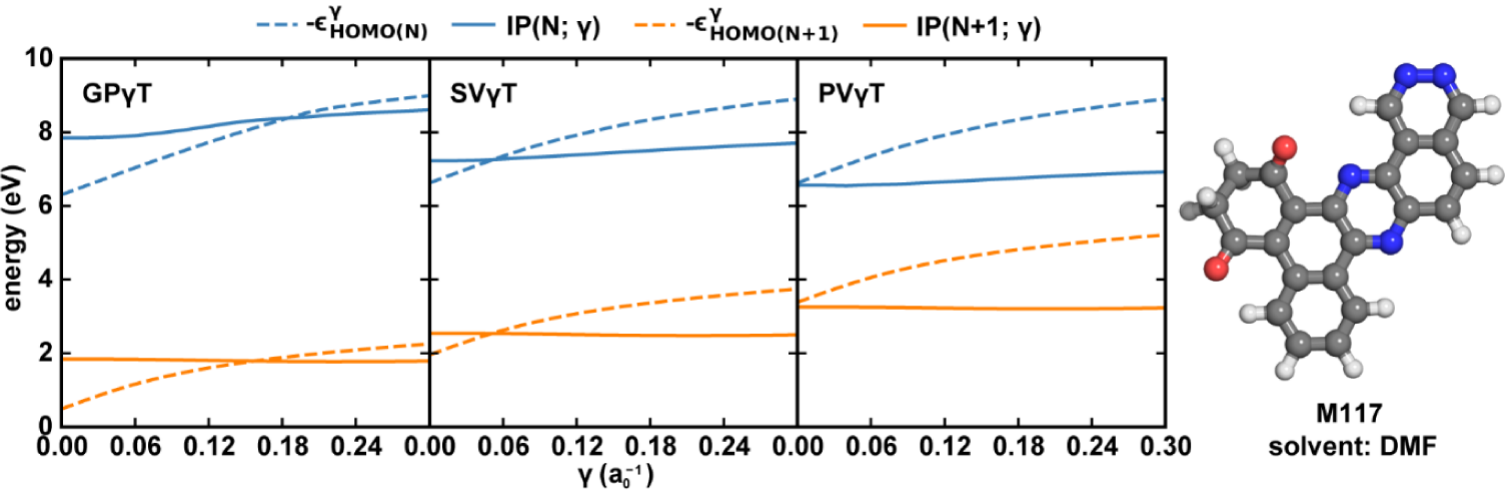
HOMO energies (dotted lines) and IP (solid lines) as a function of γ for M117 across three γ-tuning schemes. The N-electron neutral state is represented in blue, while the *N* + 1 anionic state is represented in orange. The molecular structure of M117 is depicted adjacent to the panels. PCM dielectric constants for DMF (ε = 36.7, ε_∞_ = 2.04) were applied in SVγT and PVγT calculations.

However, the trend is the opposite for the anionic state with *N* + 1 electrons. As the tuning scheme switches from GPγT to SVγT and PVγT with an increasing level of solvent relaxation applied, both $-{\text{ϵ}}_{\mathrm{HOMO}\left(N+1\right)}^{\gamma }$ and IP­(*N* + 1; γ) increases, but the increase for $-{\text{ϵ}}_{\mathrm{HOMO}\left(N+1\right)}^{\gamma }$ is more significant. Undoubtedly, the positive PCM polarization charges stabilize the anionic *N* + 1 state, decreasing both *E*(*N* + 1; γ) and ${\text{ϵ}}_{\mathrm{HOMO}\left(N+1\right)}^{\gamma }$ , as observed in charge-separated peptides.^103^ However, this does not explain why $-{\text{ϵ}}_{\mathrm{HOMO}\left(N+1\right)}^{\gamma }$ is more sensitive to the PCM field than IP­(*N* + 1; γ) = *E*(*N*; γ) – *E*(*N* + 1; γ). We believe the major reason here is that PCM’s impact on *E*(*N* + 1; γ), the total energy of the solute–solvent supersystem, includes the self-energy of the positive polarization charges and the work required to induce them.^80^ These two contributions partially offset PCM’s stabilization effect on the total energy of the anionic state, but do not offset PCM’s stabilization of the HOMO energy. This results in a less pronounced PCM impact on IP­(*N* + 1; γ) than $-{\text{ϵ}}_{\mathrm{HOMO}\left(N+1\right)}^{\gamma }$ . A more detailed mathematical proof can be found in Text S1.

To assess whether this trend holds across other systems, we select all 240 entries using DCM as the solvent (ε = 8.93, ε_∞_ = 2.03), since it is the most frequently occurring solvent in our dataset. For each entry, with γ-values fixed at 0.20 a_0_^–1^, the changes in IP­(*N*; γ), IP­(*N* + 1; γ), $-{\text{ϵ}}_{\mathrm{HOMO}\left(N\right)}^{\gamma }$ , and $-{\text{ϵ}}_{\mathrm{HOMO}\left(N+1\right)}^{\gamma }$ (Figure S6) are computed. Across all 240 entries, at the neutral state, PCM with ε = 8.93 exerts a negligible impact on $-{\text{ϵ}}_{\mathrm{HOMO}\left(N\right)}^{\gamma }$ (+0.07 eV) but significantly lowers the IP­(*N*; γ) (−1.19 eV). However, in the anionic state, the same PCM has a substantially stronger effect on $-{\text{ϵ}}_{\mathrm{HOMO}\left(N+1\right)}^{\gamma }$ (+2.66 eV) than IP­(*N* + 1; γ) (+1.39 eV). This indicates that the influence of PCM on these physical quantities across different molecules is qualitatively consistent with the behavior observed in system M117.

It is worth noting that this trend holds only if $-{\text{ϵ}}_{\mathrm{HOMO}\left(N\right)}^{\gamma }$ and $-{\text{ϵ}}_{\mathrm{HOMO}\left(N+1\right)}^{\gamma }$ increases faster with γ than IP­(*N*; γ) and IP­(*N* + 1; γ). According to Figure 4, this condition is met with system M117. This trend has been documented in numerous studies across various contexts involving γ-tuning^58,104,105^ and can be attributed to the concave-down fractional charge behavior observed with an increasing Fock exchange fraction.^29^ To verify the generality of this observation, we fit the slopes of these four quantities relative to γ for each of the 240 molecules with DCM as the solvent (Figure S7). The results indicate that $-{\text{ϵ}}_{\mathrm{HOMO}\left(N\right)}^{\gamma }$ and $-{\text{ϵ}}_{\mathrm{HOMO}\left(N+1\right)}^{\gamma }$ increase rapidly with γ exhibiting average slopes of 10.16 eV·a_0_ and 8.56 eV·a_0_, respectively. Moreover, their growth rate surpasses that of IP­(*N*; γ) and IP­(*N* + 1; γ), which have average slopes of 1.31 eV·a_0_ and −0.49 eV·a_0_, respectively.

To examine how the optimal γ varies with the solvent dielectric constant (ε), the dataset was partitioned by solvent type. We refer to entries sharing the same solvent as solvent groups (e.g., the methanol group encompasses all entries where methanol was used as the solvent). The number of entries per solvent group ranged from 44 (toluene group) to 240 (DCM group). For each solvent group, we computed the mean optimal γ-value and its standard deviation (Figure 5). Our result indicates that the optimal γ-values obtained by GPγT yield optimal γ-values largely independent of solvent polarity, since the solvent is not involved in the γ-tuning procedure. In PVγT, the optimal γ decreases with increasing ε and approaches zero for polar solvents. This trend arises because PCM’s impact on $-{\text{ϵ}}_{\mathrm{HOMO}\left(N\right)}^{\gamma }$ , $-{\text{ϵ}}_{\mathrm{HOMO}\left(N+1\right)}^{\gamma }$ , IP­(*N*; γ) and IP­(*N* + 1; γ) scales with (ε – 1)/ε, resulting in smaller optimal γ-values for polar solvents. SVγT’s optimal γ-values, however, are close to that of PVγT for nonpolar solvents, but deviate quickly when ε increases. In addition, optimal γ from SVγT shows no significant dependence on solvent polarity. This is because when applying nonequilibrium solvation to the solute’s electron addition and removal, the impact of polar solvents on $-{\text{ϵ}}_{\mathrm{HOMO}\left(N\right)}^{\gamma }$ , $-{\text{ϵ}}_{\mathrm{HOMO}\left(N+1\right)}^{\gamma }$ , IP­(*N*; γ) and IP­(*N* + 1; γ) are approximately equivalent to that of a nonpolar solvent with ε = ε_∞_ (Text S2). As all 9 solvents’ ε_∞_ values are between 2 and 2.5, their optimal γ values differ negligibly. An explanation from the physical perspective of the difference of optimal γ between PVγT and SVγT is the result of “error compensation”. PVγT inappropriately includes the solvent’s nuclear degree of freedom in the solute’s instantaneous ionization process, causing more deviation from Janak’s theorem. Given that the optimal γ decreases with ε for a given system, to compensate for this error, the optimal tuning procedure yields a smaller γ to enforce the piecewise linearity, thereby explaining the difference between PVγT and SVγT in protic solvents.

**Figure 5:**
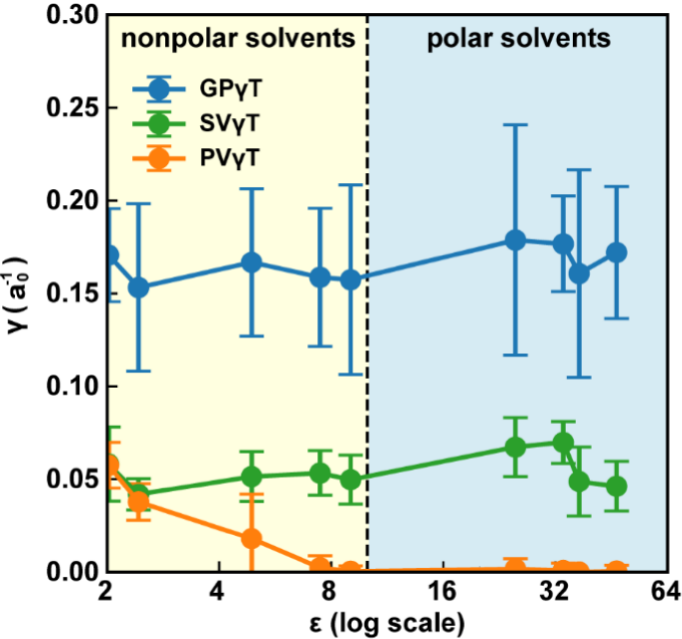
Optimal γ-values as a function of solvent dielectric constant (ε) for different γ-tuning schemes (GPγT, SVγT, PVγT). Data points represent solvent-group averages, with error bars indicating one standard deviation within each group. The dashed vertical line separates nonpolar and polar solvents.

Based on the above analysis, we conclude that the magnitude of the negative of HOMO energies increases with γ, and this increase is significantly greater than that of IP. In the neutral *N*-electron state, PCM has a negligible effect on $-{\text{ϵ}}_{\mathrm{HOMO}\left(N\right)}^{\gamma }$ but significantly reduces IP­(*N*; γ), shifting their intersection to smaller γ-values. For the anionic state, both $-{\text{ϵ}}_{\mathrm{HOMO}\left(N+1\right)}^{\gamma }$ and IP­(*N* + 1; γ) increase due to PCM; however, the change in $-{\text{ϵ}}_{\mathrm{HOMO}\left(N+1\right)}^{\gamma }$ is more pronounced, further shifting their intersection to the left. As both the neutral state and anionic state intersections shift leftward, the optimal γ progressively decreases toward zero. Thus, incorporating PCM into the γ-tuning procedure leads to a reduction in the optimal γ value, which is more pronounced in polar solvents when using PVγT. SVγT disregards the relaxation of the solvent’s slow (nuclear) degrees of freedom when computing IP and EA, thereby diminishing the impact of PCM. As the optical dielectric constant for all 9 solvents we tested here is distributed across 1.77 (methanol) to 2.24 (toluene), a small range compared with the distribution of the static dielectric constant (from 2.02 to 46.71), the optimal γ in SVγT remains slightly larger than that in PVγT and does not correlate with the solvent’s polarity.

### 3.2 Assessing the γ-Tuning Procedure

To evaluate the performance of the three γ-tuning schemes, we use the optimum γ-values obtained by GPγT, PVγT, and SVγT, together with the default γ-value of 0.2 a_0_^–1^ in the ωPBEh functional, to compute the absorption spectrum using TDDFT with TDA. Following the procedure described in Section 2.3, we focus solely on comparing the absorption energy of the first visible peak (Δ*E*_peak_) between the simulated and experimental spectra. The accuracy of each γ-tuning scheme is quantitatively assessed using mean absolute error (MAE) and mean signed deviation (MSD), computed according to eqs 6 and 7:

6 $$MAE=\frac{1}{n}\overset{n}{\underset{i=1}{\sum }}\left|\Delta E_{\mathrm{peak},i}^{\mathrm{pred}}-\Delta E_{\mathrm{peak},i}^{\exp}\right|$$

7 $$MSD=\frac{1}{n}\overset{n}{\underset{i=1}{\sum }}\left(\Delta E_{\mathrm{peak},i}^{\mathrm{pred}}-\Delta E_{\mathrm{peak},i}^{\exp}\right)$$

Here, *n* refers to the number of entries in the dataset. $\Delta E_{\mathrm{peak},i}^{\mathrm{pred}}$ and $\Delta E_{\mathrm{peak},i}^{\exp}$ are the TDDFT predicted and experimental Δ*E*_peak_ for the *i*th entry, respectively. MAE accounts for both random and systematic deviations between theoretical and experimental results, whereas MSD specifically quantifies systematic bias within different γ-tuning schemes.

As shown in Figure 6, the default γ systematically overestimates the Δ*E*_peak_, with an MSD of 0.56 eV. Applying GPγT reduces the MSD to 0.43 eV, only offering a minor improvement. The integration of PCM with the γ-tuning, however, remarkably improves the accuracy. Specifically, PVγT and SVγT lower the MSD to −0.01 and 0.04 eV, respectively. They also achieve substantially smaller MAEs, 0.36 eV for PVγT and 0.35 eV for SVγT, compared with GPγT (0.67 eV) and the default γ (0.56 eV). Because our simulations neglect vibrational structure, conformational sampling, and explicit solvent effects, the residual errors in Δ*E*_peak_ cannot be fully eliminated; indeed, even high-level wave function-based methods such as CC2 and CASPT2 exhibit MAEs of 0.20–0.30 eV for predicting experimental absorption spectra.^106^ Reoptimizing the molecular geometry with the optimal γ derived from each scheme and recalculating their absorption energy revealed that the effect of geometry relaxation is negligible (Figure S8). Furthermore, disabling the TDA in SVγT-based TDDFT calculations altered the MAE by no more than 0.01 eV (Figure S9). The result above indicates that the γ-tuning schemes with solvent effects (PVγT and SVγT) yield better optimum γ-values for predicting the solution-phase absorption spectrum.

**Figure 6:**
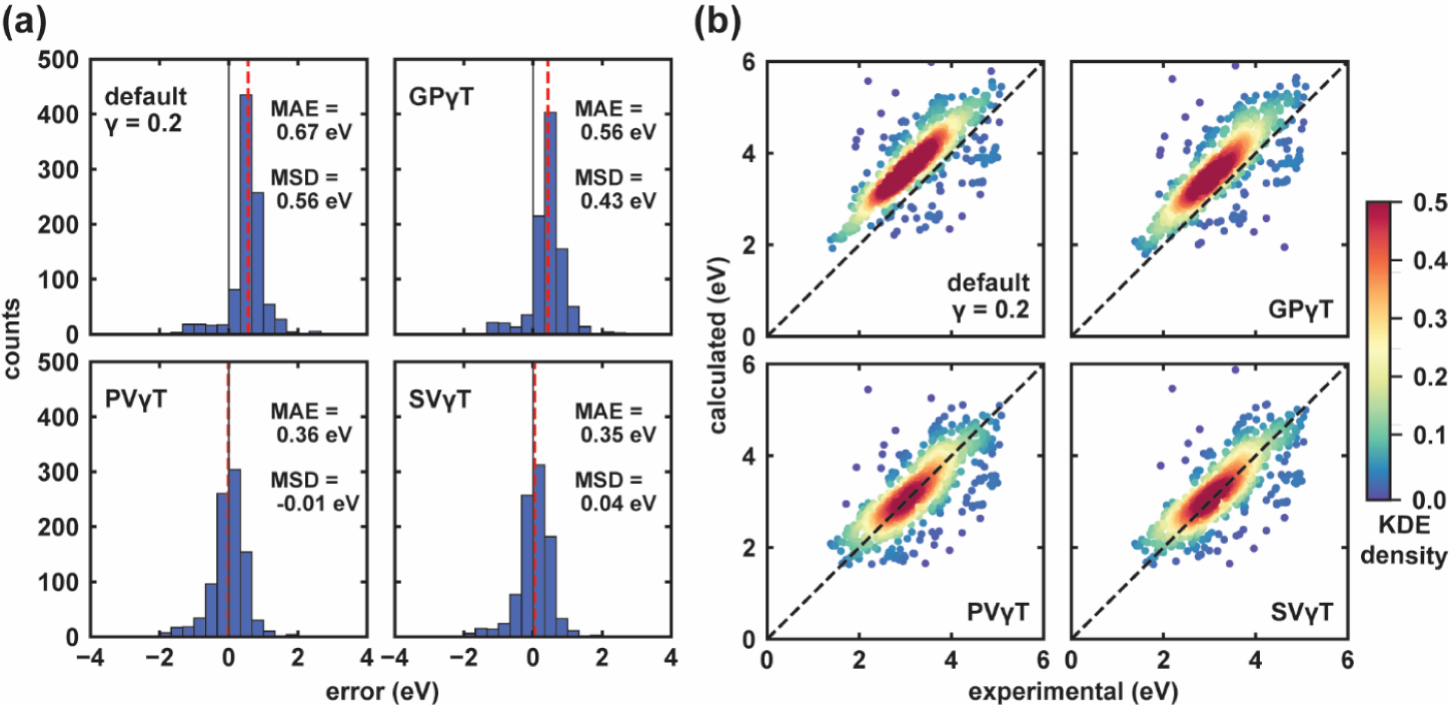
Comparative analysis of experimental and calculated Δ*E*_peak_ obtained from different γ-tuning schemes: the default γ, the GPγT, the PVγT, and the SVγT. (a) Histogram plots illustrate the distribution of prediction errors for each γ-tuning scheme. The MSD is indicated by a dashed red line, while a vertical reference line marks the ideal predictive performance (zero error). (b) Parity plots compare experimental and calculated Δ*E*_peak_ across different γ-tuning schemes. Data point densities are color-coded based on Kernel Density Estimation (KDE) values, as shown in the right color bar. The ideal agreement between experimental and predicted values is represented by diagonal dashed lines, denoting the parity line.

Interestingly, our tuned ωPBEh results do not indicate that very small γ values (primarily obtained from PVγT) lead to a severe underestimation of the excitation energies, which was one of the major concerns about the PVγT scheme.^51^ Previous benchmark studies on TDDFT calculations for singlet excited states indicate that RSH functionals tend to overestimate the local-excited (LE) state energy by 0.21–0.43 eV without γ-tuning,^28,42^ while global hybrid GGA functionals containing 20–25% exact exchange, such as PBE0,^107^ exhibit the lowest MAE in predicting the LE state energy of organic molecules.^108,109^ Notably, ωPBEh with the default α and a small γ behaves similarly to a global hybrid functional featuring 80% PBE exchange and 20% global HF exchange, closely resembling PBE0, which employs 25% global HF exchange. Hence, a small γ improves the prediction accuracy of LE states. For excitations with strong CT characters in the solvent, a small γ is also sufficient to reproduce the correct asymptotic behavior, as we will elaborate on in Section 3.4.

We also evaluated the SRSH-PCM approach on our dataset, which was originally proposed for predicting optical gaps in organic molecular crystals.^62^ In the solid state, the surrounding molecules’ geometry is fixed, and the electrostatic response can be effectively modeled by only considering the environment’s electron degree of freedom (ε_∞_). In contrast, solvent molecules can reorient in response to electron addition and removal of the solutes, giving rise to two distinct dielectric constants: ε and ε_∞_. Therefore, we performed two variants of SRSH-PCM that enforce α + β = 1/ε or α + β = 1/ε_∞_ respectively. This was done by adjusting β while fixing α as the default value (α = 0.2 for ωPBEh). To assess the performance of SRSH-PCM in the solution phase, we carried out TDDFT calculations of all 937 solutes using both variants of SRSH-PCM, with nonequilibrium PCM turned on to mimic the solvent’s response to electron excitation (Figure S10). When α + β = 1/ε, SRSH-PCM outperforms default γ and GPγT by effectively removing the systematic error, yielding a small MSD of 0.03 eV, although the MAE (0.40 eV) was slightly larger than that of SVγT. In the latter case, where α + β = 1/ε_∞_, SRSH-PCM slightly overestimates the excitation energies (MSD = 0.25 eV) and produces a higher MAE (0.42 eV) than SVγT. Overall, these results suggest that while SRSH-PCM provides a reasonable description of solvent screening, SVγT offers a slightly better accuracy for predicting solution-phase absorption spectra with ωPBEh.

We also considered adjusting both α and γ simultaneously to find the best possible (α, γ) combination. However, this requires systematic exploration of the two-dimensional parameter space, significantly increasing the computational effort and making it unsuitable for high-throughput calculations. Therefore, we cannot verify the accuracy of related approaches on the entire dataset. However, to explore the highest accuracy that can be reached by adjusting α and γ, we randomly selected 21 molecules out of the DCM group, then performed the solvation-mediated tuning procedure (sol-med-OT) proposed by Joo et al.^110^ to find the optimal α and γ values (α* and γ*). A detailed methodology described here is available in Text S3 and Figure S11. Our results show that sol-med-OT shows a systematic overestimation of 0.31 eV under the condition α + β = 1/ε_∞_ (Table S1 and Figure 7). In contrast, SVγT gives a much lesser MSD of 0.06 eV over the 21 molecules. Our 2D sweep across the parameter space (see Figure 8 for an illustrative example of M319) indicates that the α, γ pairs that make TDDFT consistent with experimental values are generally within the region of α < 0.2 and γ < 0.2 a_0_^–1^ for both α + β = 1 and α + β = 1/ε_∞_. The α* and γ* from sol-med-OT, however, fall between 0.30 and 0.40 and 0.10–0.15 a_0_^–1^, which is outside the optimal region. In contrast, γ* obtained from SVγT when α < 0.2 fall within this region, leading to a better accuracy. Considering the computational overhead of systematically investigating the 2D parameter space, we believe SVγT is an economical and accurate solution for predicting solution-phase excitation energy in high-throughput calculations.

**Figure 7:**
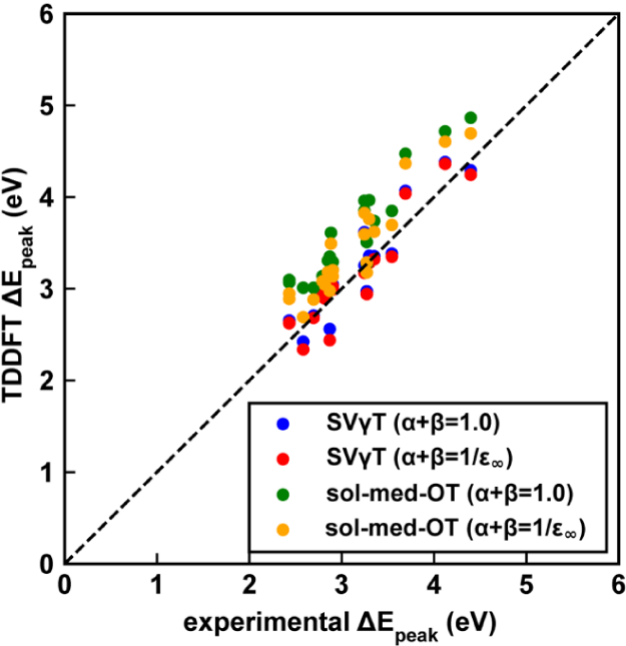
Correlation between calculated and experimental excitation energies for 21 molecules in DCM. Scatter plots compare TDDFT excitation energies obtained with different tuning strategies against experiment: SVγT with α + β = 1.0 (orange), SVγT with α + β = 1/ε_∞_ (green), sol-med-OT with α + β = 1.0 (blue), and sol-med-OT with α + β = 1/ε_∞_ (red). The dashed diagonal line indicates perfect agreement.

**Figure 8:**
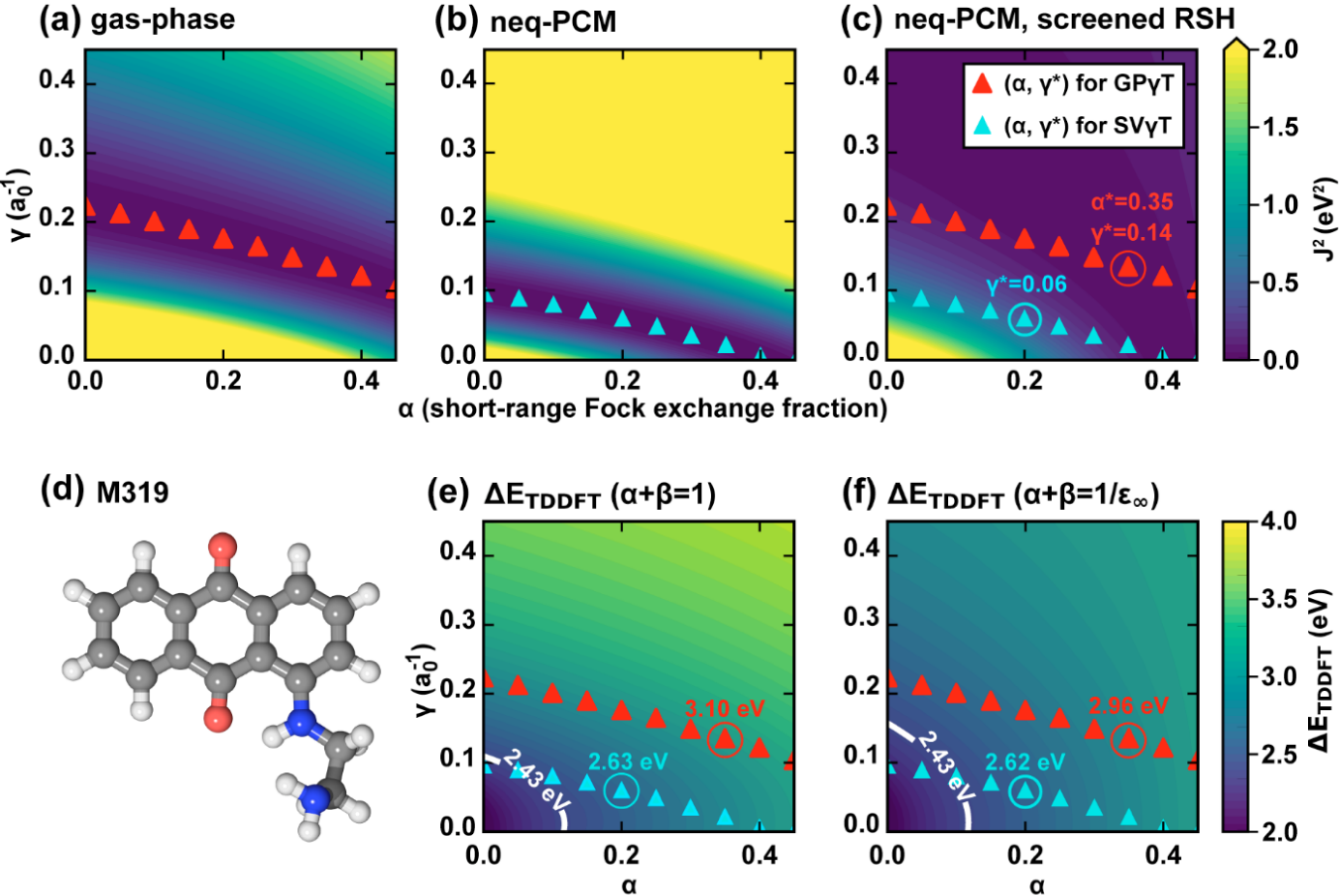
Illustration of two-parameter tuning of short-range Fock exchange fraction (α) and range-separation parameter (γ) for molecule M319 in DCM (ε = 8.96, ε_∞_ = 2.03). (a–c) Contour plots of the target function *J* ^2^(α, γ) evaluated under three conditions: (a) gas-phase (α + β = 1, ε_∞_ = 1.0), (b) nonequilibrium solvation (neq-PCM) with unscreened RSH functional (α + β = 1), and (c) nonequilibrium solvation with screened RSH functional (α + β = 1/ε_∞_). Red triangles indicate optimal γ* obtained from GPγT for each α, while cyan triangles correspond to that from SVγT. Red and cyan circles denotes the α and γ (in a_0_^–1^) obtained through sol-med-OT and SVγT, where SVγT uses the default γ = 0.2 a_0_^–1^. (d) The chemical structure of M319, with H, C, N, and O atoms colored in white, gray, blue, and red, respectively (e-f) TDDFT S_0_-S_1_ excitation energies (Δ*E*_TDDFT_) as functions of (α, γ) for unscreened (α + β = 1) (f) and screened (α + β = 1/ε_∞_) (g) ωPBEh, overlaid with the corresponding GPγT and SVγT (α, γ*) pairs. The final TDDFT excitation energy for sol-med-OT and SVγT are labeled as red and blue numbers in eV, respectively. A white contour line indicates the experiment measured excitation energy. The Δ*E*_peak_ of M319 comes solely from its S_0_-S_1_ transition.

Considering that the optimal α usually falls between 0.0 and 0.2, and the accuracy of GPγT, PVγT, and SVγT may also be improved by selecting a different α, we reperformed the three γ-tuning procedures on all 937 entries with α = 0.00, 0.05, and 0.10 (Figure 9). Notably, ωPBEh degenerates to LC-ωPBE when α = 0.00.^111^ The optimal γ values obtained by all schemes decreased as α increased (Figure S12), consistent with the observation of previous studies.^110^ For all tested α values, using default γ and GPγT constantly leads to systematic overestimation. PVγT systematically underestimates the excitation energy when α = 0.00 and 0.05 (MSD = −0.33 and −0.22 eV), possibly due to the delocalization error introduced by both α and γ being very small (γ < 0.05 a_0_^–1^). But this systematic underestimation is reduced when introducing sufficient SR Fock exchange by setting α = 0.2. SVγT, on the other hand, has the smallest MAE and MSD, with the largest error for α=0.0, where MAE = 0.39 eV and MSD = −0.15 eV. This result further proves the reliability of SVγT at different SR Fock exchange fractions.

**Figure 9:**
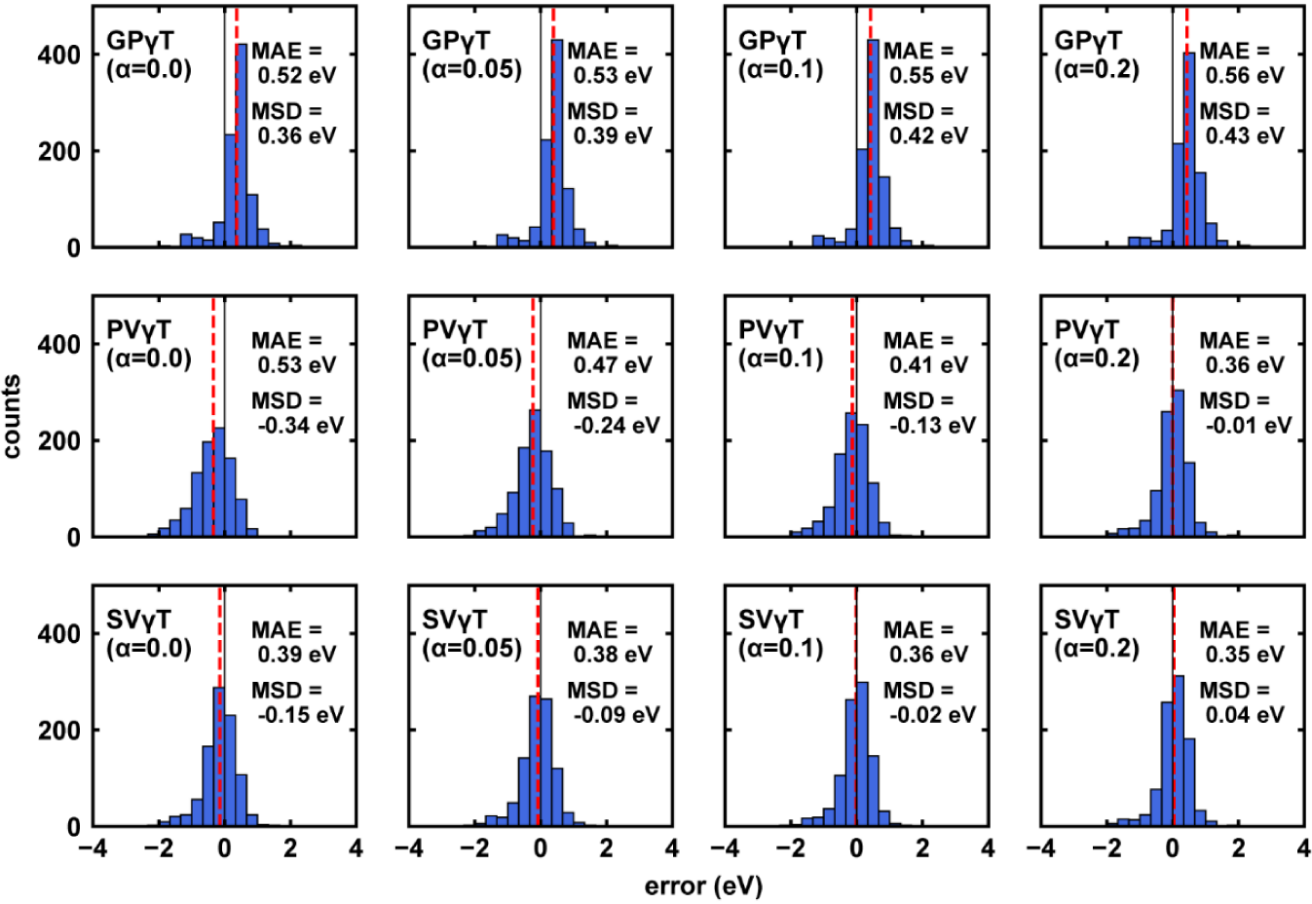
Prediction error of different γ-tuning schemes with different α. The mean γ-values for each scheme are shown in vertical dashed lines. The MAE and MSD are denoted in each panel.

Another important metric for evaluating the validity of γ-tuning for a specific molecule is the compliance with the one-particle picture, i.e., whether $\Delta \rho \left(\mathrm{r⃗}\right)={|\phi_{\mathrm{HOMO}}^{N}\left(\overset{⇀}{r}\right)|}^{2}$ holds after the γ-tuning process. We compare the R^2^ between the atomic Hirshfeld population of Δρ­(*r⃗*) and ${|\phi_{\mathrm{HOMO}}^{N}\left(\overset{⇀}{r}\right)|}^{2}$ (Figure S13). The three γ-tuning methods, GPγT, PVγT, and SVγT, have 814, 874, and 882 solutes with *R* ^2^ > 0.90, respectively. This indicates that most molecules in our dataset (937 data points) conform to the one-particle picture after γ-tuning, with SVγT having the highest compliance rate. Molecules with thiophene rings are less likely to comply with the one-particle picture (Figure S14), which can be seen from the difference between ${|\phi_{\mathrm{HOMO}}^{N}\left(\overset{⇀}{r}\right)|}^{2}$ and Δρ­(*r⃗*) of a single thiophene ring- ${|\phi_{\mathrm{HOMO}}^{N}\left(\overset{⇀}{r}\right)|}^{2}$ is localized on the sp2 carbons while Δρ­(*r⃗*) localized on the sulfur atom (Figure S15). We selected 779 out of 937 entries with all three γ-tuning schemes in compliance with the one-particle picture and recalculated their MAE and MSD (Figure S16). The results show that removing the systems that violate the one-particle picture does not affect the assessment of each γ-tuning scheme, with the change in their MAE and MSD being less than 0.02 eV.

Finally, we consider the possibility of bypassing the γ-tuning procedure altogether by directly adopting fixed γ values. Our results suggest that a small γ within 0.00 to 0.10 a_0_^–1^ may provide the most accurate performance. Accordingly, we recalculated absorption energies for all 937 entries using γ = 0.00, 0.05, and 0.10 a_0_^–1^, while fixing α = 0.20 and enforcing α + β = 1 (Figure S17). The best candidate is γ = 0.05 a_0_^–1^, yielding the lowest MAE of 0.36 eV, which is close to that obtained with SVγT (0.35 eV). The second candidate, γ = 0.00 a_0_^–1^, gives an MAE of 0.37 eV and an MSD of 0.06 eV. In this case, the RSH functional ωPBEh reduces to a global hybrid with 20% exact exchange, closely resembling PBE0, the functional benchmarked as one of the most reliable for predicting excitation energies of neutral organic molecules across more than 500 systems.^28^ Although the accuracy of fixed γ values is slightly lower than that of SVγT, adopting a small γ (e.g., 0.05) provides a computationally efficient alternative for calculating absorption spectra of organic molecules in solution.

So far, we corroborate several earlier observations based on our large and diverse dataset: PVγT and SVγT yield much smaller γ than GPγT,^55,59^ GPγT brings only minor improvement,^38^ and SRSH-PCM significantly outperforms GPγT^65^ but is slightly worse than SVγT. Our results are close to those of Sachse et al.,^60^ who found little difference between PVγT and SVγT when using ωPBEh to predict organic molecules’ UV/vis spectrum; However, PVγT is slightly inferior to SVγT in predicting Δ*E*_peak_ when using the default α = 0.20 according to our result. The common concern that PVγT’s zero γ causes underestimation in the optical gap^57–59^ appears only when the SR Fock exchange fraction is very small (e.g., α ≈ 0). This is consistent with studies done by Bokareva et al.^66^ and de Queiroz et al.,^58^ who observed systematic underestimation using range-separated functionals with α=0. Conversely, using a small γ in solution effectively emulates the dielectric screening captured by SRSH-PCM,^53,62^ which has been demonstrated by Sun et al. in organic crystals;^61^ the mechanism is analyzed later (Section 3.4). Thus, most of our findings directly connect to prior studies, while the scale and diversity here provide statistically robust confirmation and generalization beyond earlier small benchmarks.^61,66,67^

To summarize, we attach a table to visually show the MAE and MSD of all tested methods for a direct comparison (Table 1). TDDFT results using 2 untuned external DFT functionals, CAM-B3LYP^54^ and ωB97X-D3,^112^ are also attached for cross-functional comparison. Our results show that SVγT shows the optimal performance, followed by PVγT and fixed γ = 0.05 a_0_^–1^. Untuned RSH functionals, however, always overestimate the Δ*E*_peak_. Among them, CAM-B3LYP shows the lowest MSD of 0.43 eV as its LR Fock exchange fraction is 0.65 instead of 1.0, simulating the dielectric screening effect produced by the SRSH-PCM approach. However, the error reduction caused by adjusting α + β is less pronounced compared to directly tuning the range separation parameter γ with PCM. Considering the significant computational overhead for tuning α and γ simultaneously, we believe that for the prediction of Δ*E*_peak_ using ωPBEh for neutral organic solvents, SVγT is the optimal methodology.

**Table 1:** MAE and MSD in eV for All Methods We Evaluated in This Section^a^

method	MAE (eV)	MSD (eV)
γ = 0.20 a_0_^–1^ (default γ)	0.67	0.56
GPγT	0.56	0.43
PVγT	0.36	–0.01
SVγT	0.35	0.04
SRSH (α + β = 1/ε)	0.40	0.03
SRSH (α + β = 1/ε_∞_)	0.42	0.25
γ = 0.00 a_0_^–1^	0.37	0.06
γ = 0.05 a_0_^–1^	0.36	0.10
γ = 0.10 a_0_^–1^	0.42	0.26
CAM-B3LYP*	0.56	0.43
ωB97X-D3*	0.75	0.66

a All methods utilized ωPBEh functional except those labeled with an asterisk (*).

### 3.3 Impact of Solutes and Solvents

To further identify solute–solvent combinations that benefit most from γ-tuning, we analyze the relationship between solute chemical structures and TDDFT calculation errors for Δ*E*(S_1_) using the optimal γ values obtained from different tuning schemes. Additionally, we examine the role of solvents in impacting the TDDFT calculation’s performance.

We begin with a t-distributed stochastic neighbor embedding (t-SNE) analysis of the full dataset of 937 solutes. Each solute is first embedded into 1024-bit Morgan fingerprints with a radius of 2 using RDKit,^113^ followed by t-SNE analysis with cosine distance metrics implemented via the scikit-learn Python package.^114^ Subsequently, all solutes are mapped onto a two-dimensional scatter plot (Figure 10), where individual solute molecules, represented as dots, are arranged based on their structural similarities.

**Figure 10:**
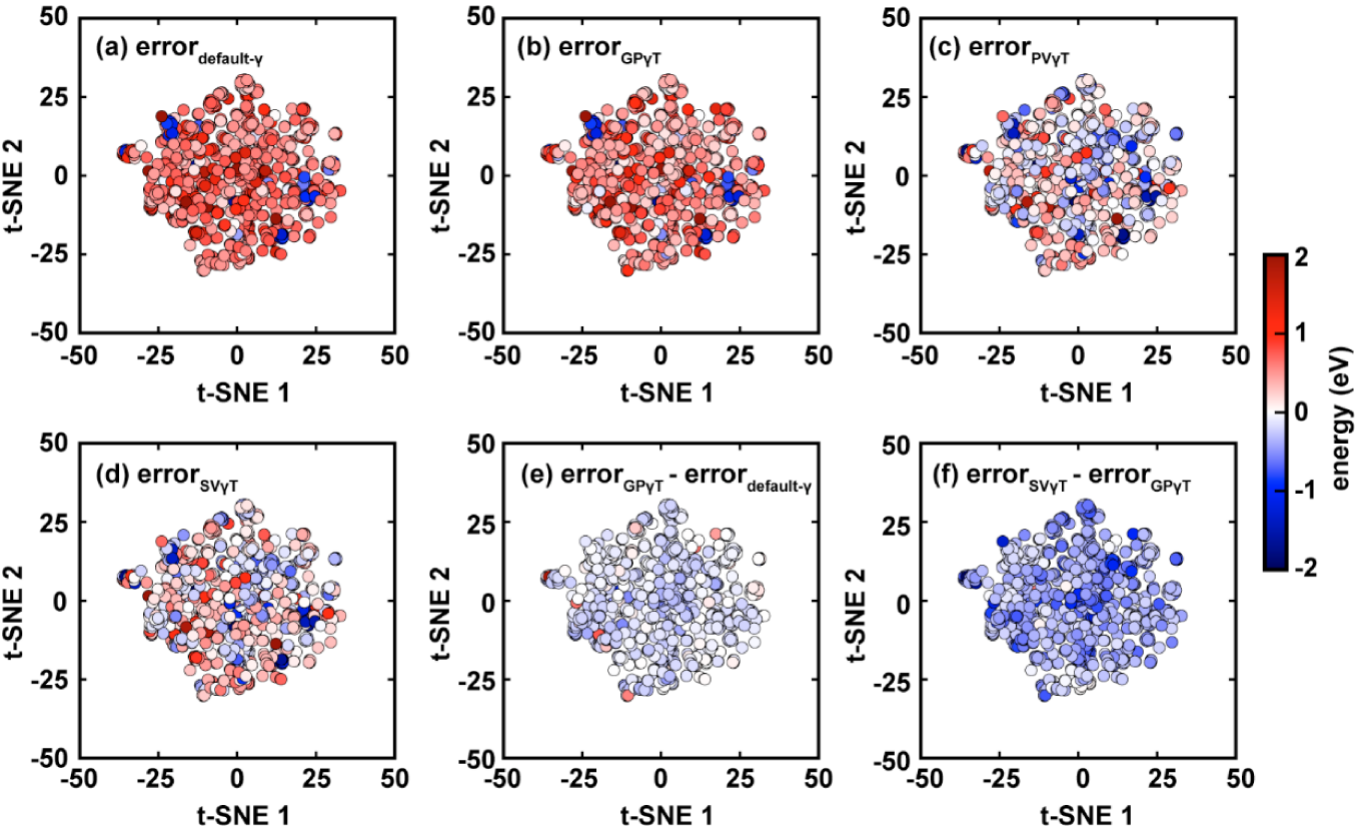
t-SNE analysis comparing prediction errors across different γ-tuning schemes. Each dot represents a solute in the dataset, arranged according to chemical similarity. Subplots a–d show the signed prediction errors (in eV) for default γ, GPγT, PVγT, and SVγT, respectively. Subplots e and f display the error differences between GPγT and default γ, and between SVγT and GPγT. The dots’ color in a–d corresponds to the signed prediction error in Δ*E*_peak_, and that in e and f is the difference in their signed error. Refer to the color bar on the right for the correspondence between color and quantities.

In subplots (a) to (d), dots are colored according to signed prediction errors, calculated by subtracting experimental Δ*E*_peak_ $\left(\Delta E_{\mathrm{peak}}^{\exp}\right)$ from the TDDFT predicted value $(\Delta E_{\mathrm{peak}}^{\mathrm{pred}}).$ Red and blue dots indicate overestimation and underestimation, respectively. Based on the t-SNE analysis shown in Figure 10 panel a–d, both the default γ and GPγT tend to overestimate excitation energies for most solutes, whereas PVγT and SVγT exhibit roughly equal proportions of overestimated and underestimated predictions. Comparing c and d, the inclusion of nonequilibrium solvation in γ-tuning does not have a significant impact on prediction accuracy, as the difference in the signed error between PVγT and SVγT is minimal for most solutes.

We examined the correlation between the prediction error and the heavy atom count in each solute for default γ and all 3 schemes (Figure 11 top panel). Generally, only GPγT’s error decreases with the solute’s size, while that of default γ, PVγT, and SVγT shows no notable correlation with the molecular size. We suspect that this is correlated with the decreasing trend of GPγT’s optimal γ (Figure 11 bottom panel), which has been observed in previous studies,^40^ but the reasons behind this phenomenon, to our knowledge, have not yet been studied thoroughly. Apart from the molecular size, the signed errors for all tuning schemes appear only weakly correlated with chemical structure, as there is no apparent gradient or cluster patterns between the prediction errors and the embedded coordinates on the t-SNE plots.

**Figure 11:**
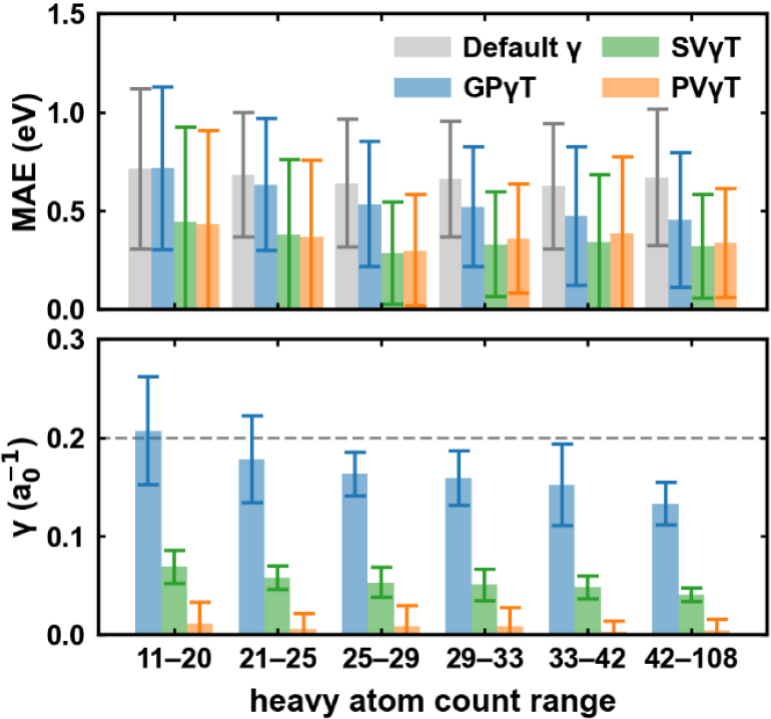
(top) Correlation between heavy atom count and MAE for default γ and different tuning schemes. (bottom) Correlation between heavy atom count and optimal γ values for different tuning schemes. The average optimal γ values are computed of GPγT, PVγT, and SVγT across solutes grouped by heavy-atom count. Error bars represent the standard deviation of the optimal γ within each group. Both panels’ *x*-axis denotes different heavy-atom count ranges, with 156 molecules in each range except the first range, which has 157 molecules.

Panels (e) and (f) of Figure 10 compare the computed excitation energy across different γ-tuning schemes. According to Figure 10 panel (e), switching from default γ to GPγT has a minor impact on $\Delta E_{\mathrm{peak}}^{\mathrm{pred}}$ , while switching to SVγT significantly reduces the value $\Delta E_{\mathrm{peak}}^{\mathrm{pred}}$ for almost all entries, thereby reducing the systematic overestimation. To further probe the structural effect, we associated the signed errors of default γ and SVγT with specific functional groups to reflect the impact of γ-tuning on the molecule’s computed excitation energy (Figure 12). Upon applying SVγT, Δ*E*_peak_ for solutes containing azo groups (−N=N−) show relatively minor change (−0.24 eV) compared to other entries (−0.58 eV), as their $\Delta E_{\mathrm{peak}}^{\mathrm{pred}}$ usually originated from the bright π–π* transition,^115^ which is relatively insensitive to the solvent’s polarity. In contrast, molecules containing nitrile groups (−C≡N) exhibit larger shifts in predicted Δ*E*_peak_ (−0.67 eV) compared to other entries (−0.51 eV), as their S_1_ state often has charge-transfer character due to the strong electron withdrawal effect of nitrile,^116^ making them more sensitive to solvent polarization.

**Figure 12:**
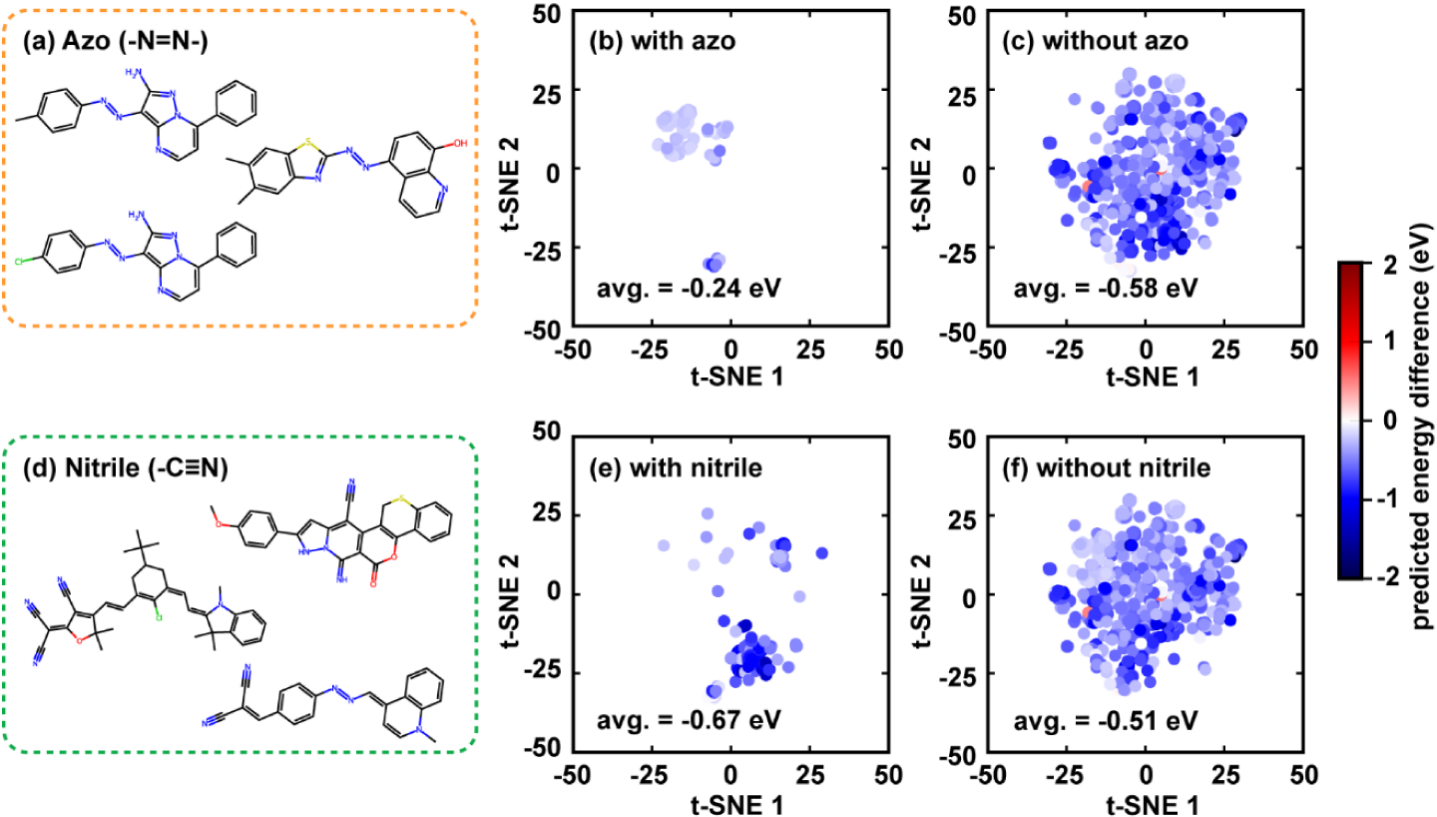
Impact of functional azo and nitrile on the difference in the predicted energy between default γ and SVγT. (a) three representative structures containing the azo functional group; (b) t-SNE coordinates of the solutes with azo group; (c) t-SNE coordinates of the solutes without the azo group; (d) three representative structures containing the nitrile group; (e) t-SNE coordinates of the solutes with nitrile group; (f) t-SNE coordinates of the solutes without the nitrile group. The dots in (b), (c), (e), (f) are colored according to the change in their calculated Δ*E*_peak_ after switching from default γ to SVγT, and they used the same embedding coordinates that were evaluated in Figure 10.

We also examined the relationship between prediction errors and the electronic state contributing most to the first observed absorption peak (Figure S18). With default γ and GPγT, excitation energies are systematically overestimated, and this tendency is particularly evident when the peak originates from S_3_ or higher excited states. In contrast, for PVγT and SVγT the state-dependent bias is largely suppressed, and no systematic overestimation is observed.

To assess solvent effects systematically, we computed the mean absolute error (MAE) for each solvent group (Figure 13 and Table 2). Results in Figure 13 align with observations in Figure 10, showing that GPγT provides only minor improvement compared to the default γ value. However, transitioning from GPγT to PVγT leads to a significant reduction in MAE across all solvent groups, with five out of nine solvent groups exhibiting an MAE decrease exceeding 0.2 eV. SVγT’s performance is comparable to PVγT across all solvent groups when using α = 0.20 and β = 0.80. The MAE of all tuning schemes shows no significant correlation with solvent polarizability, indicating that the improvement of using PVγT and SVγT is consistent regardless of the solvent’s polarity.

**Figure 13:**
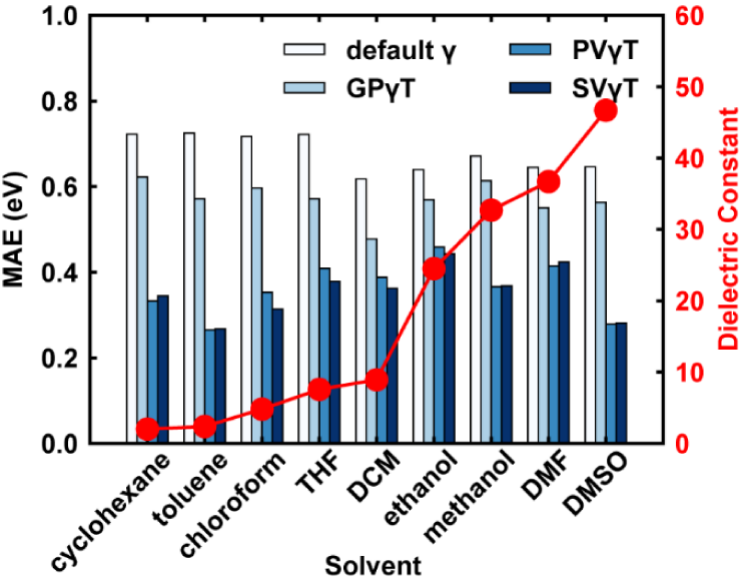
MAE of S_1_ excitation energy for different schemes of different solvent groups. The solvent dielectric constant is plotted as a red line.

**Table 2:** Size, Dielectric Constant (ε), and Average MAE across Different Solvent Groups for Default γ, GPγT, PVγT, and SVγT^a^

solvent	count	ε	MAE (default γ)	MAE (GPγT)	MAE (PVγT)	MAE (SVγT)
cyclohexane	47	2.02	0.72	0.62	0.33	0.34
toluene	44	2.38	0.73	0.57	0.27	0.27
chloroform	100	4.81	0.72	0.6	0.35	0.31
THF	104	7.58	0.72	0.57	0.41	0.38
DCM	240	8.93	0.62	0.48	0.39	0.36
ethanol	62	24.5	0.64	0.57	0.46	0.44
methanol	123	32.7	0.67	0.61	0.37	0.37
DMF	63	36.7	0.64	0.55	0.41	0.42
DMSO	154	46.7	0.65	0.56	0.28	0.28

a All MAEs reported here are in the unit of eV.

Although SVγT offers an efficient and affordable strategy for high-throughput calculations, accurately modeling the solvatochromism for specific systems often requires explicit solvation, particularly to capture hydrogen-bonding effects that implicit solvent models such as PCM cannot adequately describe.^117–119^ As an illustrative example, molecule M1074 exhibits an experimental absorption peak at 300 nm (4.13 eV), whereas SVγT predicts 4.30 eV. This discrepancy is reduced when several methanol molecules are added through molecular dynamics simulations (detailed procedure in Text S4). The excitation responsible for this peak is the S_0_-S_1_ transition, corresponding to a HOMO–LUMO transition with the LUMO localized on the pyridine ring of the solute (Figure S19). Upon addition of explicit methanol molecules, hydrogen bonds formed between methanol and the pyridine nitrogen, stabilizing the LUMO and thereby redshifting the predicted absorption peak to 302 nm (4.11 eV). Therefore, to accurately capture specific solute–solvent interactions, particularly in cases where hydrogen bonding plays a role, TDDFT calculations should be performed on the solute with a small number of explicit solvent molecules included after the γ-tuning process for the solute. However, fully capturing hydrogen-bonding effects on the absorption spectrum requires averaging TDDFT results over multiple MD snapshots, and the inclusion of explicit solvent molecules substantially increases computational cost. Thus, for high-throughput applications, γ-tuning combined with PCM remains a practical approach.

### 3.4 Impact of γ-Tuning on Delocalization Error

In Section 3.4, we showed that PVγT and SVγT outperform GPγT in reproducing experimental excitation energies. However, the optimal γ values obtained from PVγT are often close to 0.00 a_0_^–1^, raising concerns that such small values may reintroduce the delocalization error (DE) inherent to GGA functionals. Such concerns prompt us to study the impact of γ on the fractional charge curve under different solvation conditions.

The analysis was carried out following the procedure of Hemmingsen et al.,^120^ which precisely approximates the fractional charge curve by cubic spline interpolation. First, we calculated the energies *E*(*q*) at different fractional charges *q* using eq 8, and the corresponding delocalization error was quantified by eq 9, which was obtained by removing (Δ*E*)*q* from eq 8.

8 $$E\left(q\right)=\left(\Delta E\right)q+\left[\left(\varepsilon_{\mathrm{LUMO}\left(N\right)}-\Delta E\right)\left(1-q\right)+\left(\Delta E-\varepsilon_{\mathrm{HOMO}\left(N+1\right)}\right)q\right]q\left(1-q\right)$$

9 $$F\left(q\right)=\left[\left(\varepsilon_{\mathrm{LUMO}\left(N\right)}-\Delta E\right)\left(1-q\right)+\left(\Delta E-\varepsilon_{\mathrm{HOMO}\left(N+1\right)}\right)q\right]q\left(1-q\right)$$

Here, Δ*E* = IP­(*N* + 1), ε_LUMO(*N*)_ and ε_HOMO(*N*+1)_ are the LUMO energy for the N-electron state and the HOMO energy for the *N* + 1-electron anionic state. The *E*(*q*) and *F*(*q*) are plotted with the system transitions from the N-electron neutral state (*q* = 0) to the *N* + 1 anionic state (*q* = 1). *F*(*q*) = 0 indicates perfect piecewise linearity (no DE), while negative and positive values correspond to delocalization and over localization errors, respectively.

We carried out DFT calculations for system M314 using the DCM solvent with ε = 8.93 and ε_∞_ = 2.03. Then we evaluated the DE on M314 using the optimal γ-values found by the GPγT (0.16 a_0_^–1^), SVγT (0.06 a_0_^–1^), and PVγT (0.00 a_0_^–1^) under three different solvation conditions: (i) the gas phase, where no solvent response is included; (ii) nonequilibrium solvation, where only the fast electronic degree of freedom (ε_∞_) responds to electron addition; and (iii) equilibrium solvation, where both the electronic (ε_∞_) and nuclear (ε) degree of freedom responds. These three scenarios correspond to the physical pictures of GPγT, SVγT, and PVγT, respectively.

As shown in Figure 14, γ = 0.16 a_0_^–1^ (GPγT) minimizes the DE in the gas phase, while γ = 0.06 a_0_^–1^ (SVγT) minimizes the DE under nonequilibrium solvation, and γ = 0.00 a_0_^–1^ (PVγT) minimizes the DE under equilibrium solvation. Larger γ values in the solvated cases lead to overlocalization, whereas smaller γ values in the gas phase lead to delocalization. This behavior can be rationalized by the fact that the PCM itself can alleviate delocalization errors, with its extent increasing with the dielectric constant.^103,121^ As a result, the Hartree–Fock exchange fraction required to balance the DE of GGA decreases in solution, resulting in the smaller optimal γ from PVγT and SVγT.

**Figure 14:**
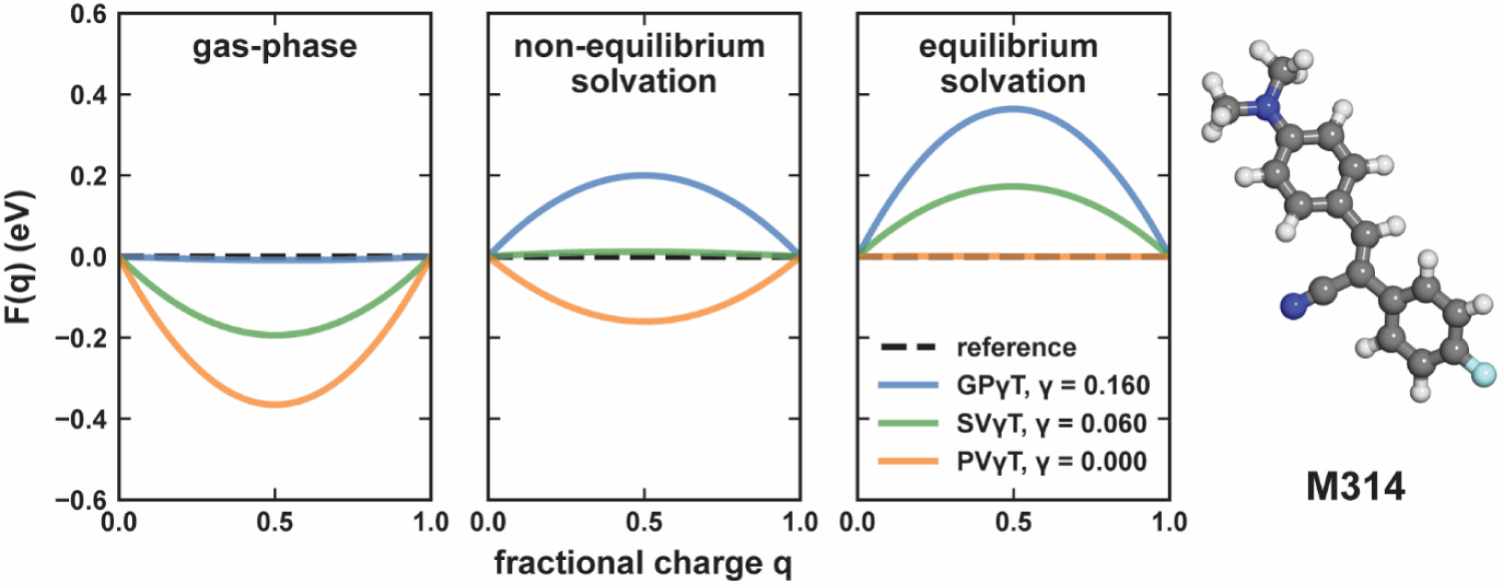
Fractional charge vs deviation from the linearity plots for M314. The deviation from linearity was estimated by eq 9, where *q* = 0 indicates the N-electron neutral state and *q* = 1 indicates the *N* + 1-electron anionic state.

Taken together, these results indicate that each γ-tuning scheme effectively minimizes the DE under the physical conditions it is designed to represent: GPγT in the gas phase, PVγT in equilibrium solvation, and SVγT in nonequilibrium solvation where only the electronic degrees of freedom respond. Thus, while very small γ values would indeed reintroduce the delocalization error in vacuum, the PCM itself compensates for this error in solution, making smaller γ values optimal. Importantly, because only the solvent’s electron degree of freedom responds to the optical absorption of the solute, which is most appropriately depicted by the nonequilibrium solvation picture. Therefore, SVγT provides the most appropriate minimization of delocalization error for modeling absorption spectra, even though its optimal γ values are typically smaller than GPγT and default γ.

### 3.5 Reasons for the Better Performance of PVγT and SVγT

We have demonstrated that, despite their small optimal γ, PVγT and SVγT can significantly improve the accuracy of solution-phase absorption energy prediction. However, the mechanism of such improvement is still unclear. Specifically, given that GGA functionals or global hybrid functionals fail catastrophically on CT states, why does a tuned RSH functional with small γ, which now behaves more like a global hybrid functional, have relatively higher prediction accuracy for the solution-phase CT excitation energies?

This question can be qualitatively answered by analyzing the asymptotic behavior in the solution phase. The advantage of RSH functionals in calculating the CT state energy can be attributed to the correct 1/*R* asymptotic behavior,^95^ where *R* can be considered as the distance between the hole and the electron. However, in the solution phase, due to the solvent’s electrostatic shielding effect as a dielectric, the correct asymptotic behvior of the Coulombic interaction between the hole and the electron becomes 1/(ε*R*), where ε represents the solvent’s dielectric constant.^62,110,122^ Therefore, the correct asymptote behavior that RSH functionals should reproduce in the solution phase becomes 1/(ε*R*) instead of 1/*R* as shown in eq 4. Since the denominator becomes larger, the HF exchange in the RSH functional must be reduced to achieve the correct asymptotic behavior. One straightforward approach is the previously mentioned SRSH-PCM procedure,^110,123^ which enforced the 1/(ε*R*) asymptotic behavior by setting the LR Fock exchange portion to 1/ε_∞_ while choosing the optimal γ without PCM. Here, we demonstrate that without limiting the LR HF exchange portion, a similar 1/(ε*R*) behavior can also be achieved by using a smaller γ value in the ωPBEh functional.

To assess the agreement between the TDDFT calculated CT state energies and the 1/(ε*R*) asymptotic behavior, we focus on a set of ethylene–tetrafluoroethylene (ETH–TFE) dimers, a well-studied system with CT excitation.^38,95^ Figure 15 compares the CT excitation energy from LR-PCM TDDFT using different γ-values $\left(\Delta E_{\mathrm{CT}}^{\mathrm{TDDFT},\gamma }\right)$ with the reference asymptotic $\Delta E_{\mathrm{CT}}^{\mathrm{asymptotic}}$ and $\Delta E_{\mathrm{CT}}^{\mathrm{ChElPG}}$ from eqs 4 and 5. In the gas phase, the default γ (0.20 a_0_^–1^) most accurately reproduces the expected asymptotic behavior. This outcome is anticipated, as one of the primary objectives in selecting the default γ for the ωPBEh functional is to reproduce the lowest CT excitation energy of the ETH–TFE dimer.^39^ However, when solvent effects are considered, the reference $\Delta E_{\mathrm{CT}}^{\mathrm{asymptotic}}$ and $\Delta E_{\mathrm{CT}}^{\mathrm{ChElPG}}$ decrease significantly due to solvent stabilization of the charge-separated excited state. Additionally, $\Delta E_{\mathrm{CT}}^{\mathrm{asymptotic}}$ and $\Delta E_{\mathrm{CT}}^{\mathrm{ChElPG}}$ exhibit a slower increase with distance compared to their gas-phase trend because of the screening effect introduced by the solvent,^62,122^ making their behavior more similar to $\Delta E_{\mathrm{CT}}^{\mathrm{TDDFT},\gamma }$ with γ = 0.10 a_0_^–1^. $\Delta E_{\mathrm{CT}}^{\mathrm{TDDFT},\gamma }$ , $\Delta E_{\mathrm{CT}}^{\mathrm{asymptotic}}$ , and $\Delta E_{\mathrm{CT}}^{\mathrm{ChElPG}}$ all show little sensitivity to solvent polarity, as the ε_∞_ values for all tested solvents are consistently around 2.1. Given that only the electronic degrees of freedom are assumed to respond to CT excitation, the impacts of the static dielectric constant on all CT energies are negligible. These results suggest that a smaller γ value can reproduce the expected 1/(ε*R*) asymptotic behavior, thereby improving the accuracy of CT excitation energy. This also explains why SVγT and SRSH-PCM share similar impacts on excitation energy: they reproduce the asymptotic behavior of 1/(ε*R*) in different ways.

**Figure 15:**
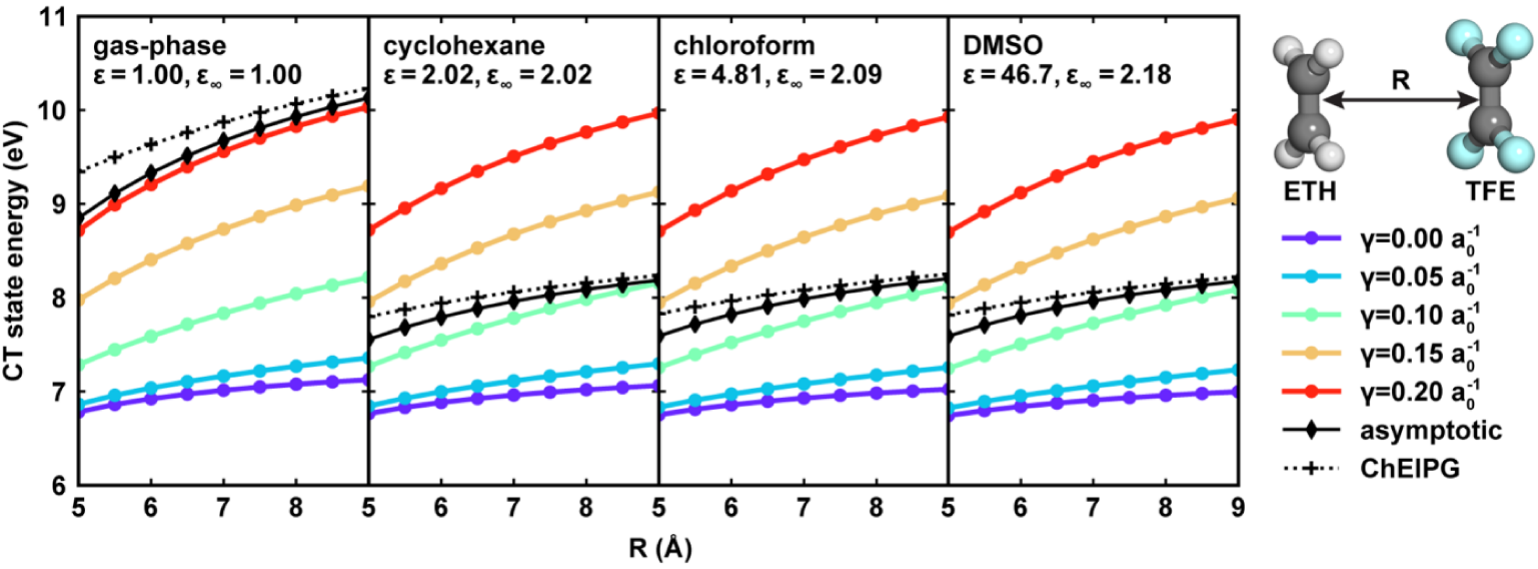
CT excitation energies of the ethylene–tetrafluoroethylene (ETH–TFE) dimer vs intermolecular distance coordinate computed with LR-PCM at different γ values in gas-phase, cyclohexane, chloroform, and DMSO. The static and optical dielectric constant for each condition is denoted in brackets. The reference curves are obtained from eqs 4 and eq 5 with the CCSD­(T) computed IP and EA.

In contrast to the significant shift of the $\Delta E_{\mathrm{CT}}^{\mathrm{asymptotic}}$ curve from gas phase to solution phase, the nonequilibrium LR-PCM TDDFT calculated Δ*E*_CT_ curves remain almost identical across all panels of Figure 15. This unexpected observation indicates that the nonequilibrium LR-PCM does not substantially impact the Δ*E*_CT_ prediction, contradicting our chemical intuition that solvent stabilizes CT states even if only the electric degree of freedom responds. This can be partially attributed to the small ε_∞_ for all solvents, but we attribute this discrepancy mainly to the LR-PCM formalism. As mentioned by Cammi et al.,^68^ the impact of LR-PCM and the state-specific PCM^48^ (SS-PCM) on excitation energy is proportional to |μ_T_|/|Δμ|, where μ_T_ represents the transition dipole moment and Δμ denotes the difference in dipole moments between the ground and excited states. Since intermolecular CT excitations typically have negligible μ_T_ but large Δμ, LR-PCM tends to underestimate the solvent response in both equilibrium and nonequilibrium solvation.

To further investigate the collaborative impact of γ and SS-PCM on the asymptotic behavior, we repeated all calculations for all ETH–TFE pairs with SS-PCM using Q-Chem. The nonequilibrium formula was used with the static and optical dielectric constant set to the corresponding solvents, with the state-tracking algorithm enabled to let the PCM equilibrate with the same CT state during external iterations (Figure 16). The results are consistent with our expectations, SS-PCM significantly lowers the CT energy for all ETH–TFE pairs, resulting in the optimal γ for reproducing the 1/(ε*R*) asymptote behavior to be 0.20 a_0_^–1^. Since the SS-PCM’s polarization charge is redistributed according to the CT excitation, the electrostatic interaction between the ETH cation and TFE anion has already been screened out by the polarization charge, so reducing γ will lead to an underestimation of CT energy. In contrast, LR-PCM does not respond to the charge redistribution, leading to zero dielectric screening effect for CT states. In this case, a small γ must be chosen to compensate for the artifact of LR-PCM and reproduce the 1/(ε*R*) behavior. Therefore, for accurately modeling the CT excitation of a particular system in solvent, SS-PCM is preferred as it more comprehensively captures the solvent’s response and rests on a firmer physical foundation. Moreover, a larger γ value should be used in conjunction with SS-PCM to avoid underestimating CT excitation energies. The above result inspired us to wonder whether we can use SS-PCM to simulate the dielectric screening effect without conducting solution-phase γ-tuning or enforcing α + β = 1/ε. Since SS-PCM requires multiple iterations to converge to the desired electronic state and requires individual calculations for each excited state included in the absorption spectrum, using SS-PCM on all 937 entries is unfeasible due to substantial computational cost. An alternative solution is using the perturbative state-specific PCM (ptSS-PCM)^124,125^ to approximate the solvent’s response to the solute’s charge redistribution. We keep α + β = 1 and use the optimal γ values from GPγT, then predict Δ*E*_peak_ using TDDFT with ptSS-PCM enabled. The calculations here were performed using Q-Chem with the same functional and basis set. However, the results are disappointing, with a systematic overestimation of 0.45 eV across the dataset (Figure S20). Since both the first absorption peak of the experimental and calculated absorption spectra contributed mostly from bright states, which typically have a larger transition dipole moment but less CT character. The ptSS-PCM effect has a lesser effect on these states, thereby cannot reduce the systematic error in GPγT. Hence, LR-PCM combined with SVγT remains an economical choice for high-throughput calculations.

**Figure 16:**
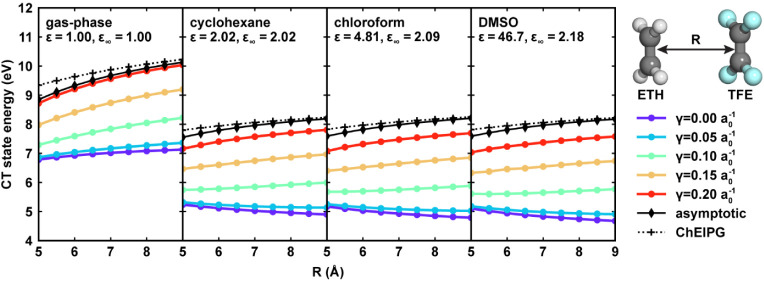
CT excitation energies of the ETH–TFE dimer vs intermolecular distance coordinate calculated using SS-PCM. The CT energy for each dimer is computed at different γ values in gas-phase, cyclohexane, chloroform, and DMSO. The static and optical dielectric constant for each condition is denoted in brackets. The reference curves are obtained from eqs 4 and 5 with the CCSD­(T) computed IP and EA.

Finally, we apply GPγT, PVγT, and SVγT to all dimer pairs solvated in cyclohexane, chloroform, and DMSO (Figure S21). Among all solvents, GPγT gives an optimal γ = 0.24 a_0_^–1^, close to the default γ = 0.20 a_0_^–1^, yet fails to give the reference Δ*E*_CT_ in solvent. The optimal γ from PVγT decreases with increasing solvent polarity, whereas that from SVγT fluctuates around 0.10 a_0_^–1^. Given that the asymptotic behavior of Δ*E*_CT_ aligns with eqs 4 and 5 when γ = 0.10 a_0_^–1^, we conclude that SVγT is the most suitable approach for describing CT excitation between ETH and TFE. This conclusion is further supported by the fact that SVγT provides a physical picture consistent with our previous assumptions. Additionally, since SVγT performs similarly to PVγT in predicting excitation energies for 937 solvated molecules with α = 0.2, but performs significantly better for smaller α values, we determine that, considering both physical reasoning and computational accuracy, SVγT is the optimal γ-tuning procedure.

## 4 Conclusion

In this work, we presented a comprehensive data-driven evaluation of three γ-tuning protocols, gas-phase γ-tuning (GPγT), partial vertical γ-tuning (PVγT), and strict vertical γ-tuning (SVγT), for predicting solution-phase absorption energies of organic molecules using the RSH functional ωPBEh under the TDDFT framework. We curated a human-reviewed benchmark dataset comprising 937 diverse neutral organic molecules with experimentally measured absorption spectra in 9 different solvents, enabling a comprehensive statistical assessment of different schemes.

The incorporation of implicit solvent effects via PCM fundamentally reduced the optimal γ values obtained through tuning: both PVγT and SVγT produce significantly lower optimal γ-values in comparison with GPγT, often close to zero for PVγT and below 0.1 a_0_^–1^ for SVγT. This phenomenon stems from the imbalanced impact of PCM on $-{\text{ϵ}}_{\mathrm{HOMO}\left(N\right)}^{\gamma }$ , $-{\text{ϵ}}_{\mathrm{HOMO}\left(N+1\right)}^{\gamma }$ , IP­(*N*; γ), and IP­(*N* + 1; γ). Specifically, PCM’s influence on IP­(*N*; γ) is greater than $-{\text{ϵ}}_{\mathrm{HOMO}\left(N\right)}^{\gamma }$ , whereas its effect on $-{\text{ϵ}}_{\mathrm{HOMO}\left(N+1\right)}^{\gamma }$ exceeds that on IP­(*N* + 1; γ). Detailed theoretical and data analyses clarify that these inequalities hold universally across all neutral organic solutes in our dataset. This effect is accentuated in PVγT, where both the nuclear and electron responses are considered, while SVγT, which considers only the electronic response, yields intermediate γ values.

In terms of practical prediction accuracy, both PVγT and SVγT offer substantial improvements in solution-phase absorption energy predictions over GPγT and the default γ value in ωPBEh. The default γ = 0.20 a_0_^–1^ and GPγT systematically overestimate excitation energies, while PCM-inclusive tuning nearly eliminates systematic bias and appreciably lowers MAE to 0.35 eV. Notably, SVγT achieves the highest compliance with the one-particle picture while having similar accuracy to PVγT. PVγT and SVγT also give slightly better accuracy than the SRSH-PCM (MAE = 0.40 eV) approach that sets the LR Fock exchange to 1/ε, while SVγT outperforms PVγT when the SR Fock exchange fraction α is smaller than 0.2. Full two-parameter (α, γ) tuning yields limited additional benefit at substantially higher cost, making it impractical for high-throughput calculation. As a pragmatic alternative, a fixed small γ (e.g., 0.05 a_0_^–1^ in ωPBEh with α = 0.20 and α + β = 1) delivers an MAE close to SVγT (∼0.36 eV) and can be attractive for very large libraries.

Further t-SNE analyses and solvent-specific error evaluations demonstrate that the improvements afforded by PVγT and SVγT are broadly applicable across chemical diversity, with little error variation that can be systematically associated with molecular structure or solvent polarity. Adding a few explicit solvent molecules after the γ-tuning procedure reduced the artifact attributable to the inability of PCM to model hydrogen bonding, offering more improvements in the accuracy for modeling the solvatochromism in protic solvents.

Mechanistically, small γ values in solution do not “reintroduce” the DE. Fractional-charge analyses show that each scheme minimizes the delocalization error in its corresponding solvation condition (gas-phase for GPγT, equilibrium PCM for PVγT, and nonequilibrium PCM for SVγT). As PCM itself partially cancels DE, thereby decreasing the required HF exchange fraction for neutralizing the DE originated from GGA functionals, naturally driving optimal γ for minimizing DE downward in the solution phase.

We also explored the physical basis for the robust performance of the tuned ωPBEh with small γ on solution-phase CT excitations. Using the ETH–TFE dimer, a classical model system, we showed that smaller γ values facilitate the correct 1/(ε*R*) asymptotic behavior required in the solution phase, in agreement with theoretical predictions and high-level ab initio calculations. Specifically, SVγT reproduced the 1/(ε*R*) asymptotic behavior with an optimal γ around 0.10 a_0_^–1^, as it provides a physical picture consistent with the nonequilibrium solvation during the electronic excitation. While state-specific PCM (SS-PCM) can be advantageous for individual, strongly CT-dominated cases, typically with a larger γ than that obtained using LE-PCM, it is computationally costly and thus ill-suited for high-throughput. Considering both physical meaning and computational accuracy, we conclude that SVγT is the optimal γ-tuning procedure for solution-phase absorption energy prediction using ωPBEh.

This study provides a large-scale, statistically grounded recommendation for γ-tuning protocols in solution-phase excited state simulations, finding SVγT as a physically robust and computationally accurate approach for tuning γ for the ωPBEh functional in UV/vis calculations of organic molecules in implicit solvent. The curated dataset can also be used to evaluate other solution-phase γ-tuning protocols. Future research should extend these insights to other RSH functionals beyond ωPBEh and to broader classes of systems, including transition metal complexes. Additionally, combining explicit solvation models or other implicit solvent models with optimal γ-tuning represents a promising direction for addressing persistent challenges in protic and highly interactive solvent environments.

## Supplementary Material





## Notes

Distribution of first-peak excited-state assignments and an example SVγT vs experiment spectrum (M472); comparisons of optimal γ from alternative loss functions and tuning accuracy diagnostics; proof of PCM’s impact on HOMO energies and IP for the *N* + 1 state, plus PCM-induced changes and γ-slopes of HOMO/IP in DCM; assessment of solvent polarity effects on optimal γ under SVγT (nonequilibrium PCM); impacts of geometry relaxation and TDA, and comparison of SRSH-PCM variants with other γ-tuning schemes; tuning workflow for sol-med-OT and result for 21 solutes in the DCM group; one-particle picture compliance tests across schemes and the influence/visualization of thiophene’s HOMO density and density difference between its neutral and cation state; performance restricted to one-particle compliant entries; accuracy of using fixed γ in ωPBEh; correlations of heavy-atom count with MAE and optimal γ; state-wise analyses of prediction error. ; explicit-solvation protocol for M1074 and its frontier orbital densities; Comparison of GPγT + ptSS-PCM vs SVγT + neq-LR-PCM across the dataset, and optimal γ values for the ETH–TFE dimers with respect to their separation distance in different solvents (PDF)
Dataset with each solute–solvent system, their S_1_ excitation energies obtained from experimentally measured UV/vis absorption spectroscopy, their optimal γ values obtained with different tuning schemes, the corresponding Δ*E*_peak_ calculated with TDDFT, the one-particle picture correlation values, sample TeraChem input for GPγT, PVγT, and SVγT, Python scripts for doing γ-tuning, and DFT optimized XYZ coordinates of all systems in the dataset (ZIP)
