# Supplementary material for: Data-Driven Recommendation of Optimal Tuning Scheme for Range-Separated Hybrid Functionals in Solution-Phase UV/Vis Absorption Energy Prediction
Source: J Chem Theory Comput. 2025 Oct 23;21(21):11106–25. doi: 10.1021/acs.jctc.5c01044 (PMC12613327; doi:10.1021/acs.jctc.5c01044)
Supplement: Supplementary file 1 [file ct5c01044_si_001.pdf]

Supporting Information for
Data-Driven Recommendation of Optimal Tuning Scheme for Range-Separated Hybrid Functionals in Solution-Phase UV/vis Absorption Energy Prediction

Fangning Ren,[#] Pinyuan Li,[#] Xu Chen, Lechen Dong, Fang Liu[*]

Department of Chemistry, Emory University, Atlanta, Georgia, 30322

Table of Contents

Figure S1. The distribution of the computed excited states is associated with the first observed absorption peak. 3

Figure S2. Comparison of the SVγT calculated and experimental spectrum for system M472. 3

Figure S3. Comparison of optimal γ values obtained using different loss functions in γ tuning procedures. 4

Figure S4. Distribution of the smallest J^2 for three tuning schemes. 4

Figure S5. GPγT result for M33. 5

Text S1. Proof of the impact of PCM on the HOMO energy and IP for the N+1 anionic state. 5

Figure S6. Change in the HOMO energies and IP after applying PCM to the DCM solvent group. 7

Figure S7. Slope of HOMO energies and IP relative to γ for the DCM solvent group. 8

Text S2. Assessing the impact of solvent polarity on optimal γ under SVγT. 8

Figure S8. Impact of geometry relaxation on the performance of different schemes. 9

Figure S9. Impact of TDA on SV 10

Figure S10. Comparison of the two variants of SRSH-PCM with other γ-tuning schemes. 10

Text S3. Procedure of tuning the short-range exact exchange fraction (α) and the range-separation parameter (γ) together. 11

Figure S11. Workflow for two-parameter tuning of (α, γ) in the sol-med-OT framework. 11

Figure S12. Distribution of optimal γ values for different γ-tuning schemes with different α. 13

Figure S13. Result for one particle picture test of different γ-tuning schemes. 13

Figure S14. Impact of thiophene on one-particle picture compliance. 14

Figure S15. Comparison of thiophene's HOMO density and electron density change after ionization. 14

Figure S16. Performance of different γ-tuning schemes only for entries in compliance with the one-particle picture. 15

Figure S17. Performance of using fixed γ values. 15

Figure S18. KDE plot of error distribution classified by the excited state associated with the simulated absorption peak. 16

Text S4. Procedure for generating the explicit solvent cluster for M1074. 16

[#] These authors contribute equally to this work
[*] Electronic mail: fang.liu@emory.edu

Figure S19. HOMO and LUMO density of solvated M1074. 17
Figure S20. Comparison of GPγT using ptSS-PCM and SVγT. 17
Figure S21. Optimal γ for the ETH-TFE dimers with respect to their separation distance in different solvents. Note, since the 18

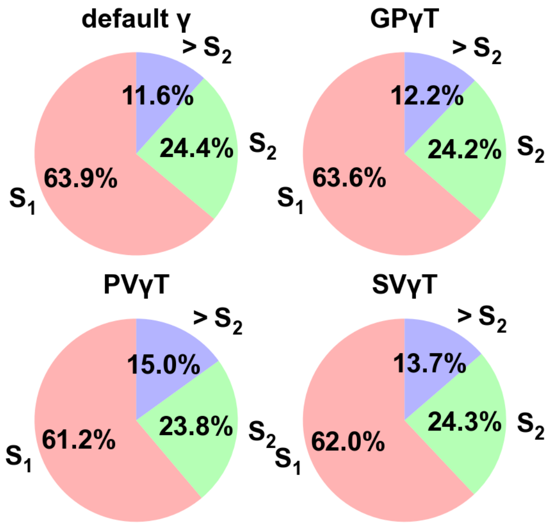

Figure S1. The distribution of the computed excited states is associated with the first observed absorption peak. Pie charts show the proportion of cases where the first visible peak originates from S_1, S_2, or higher excited states.

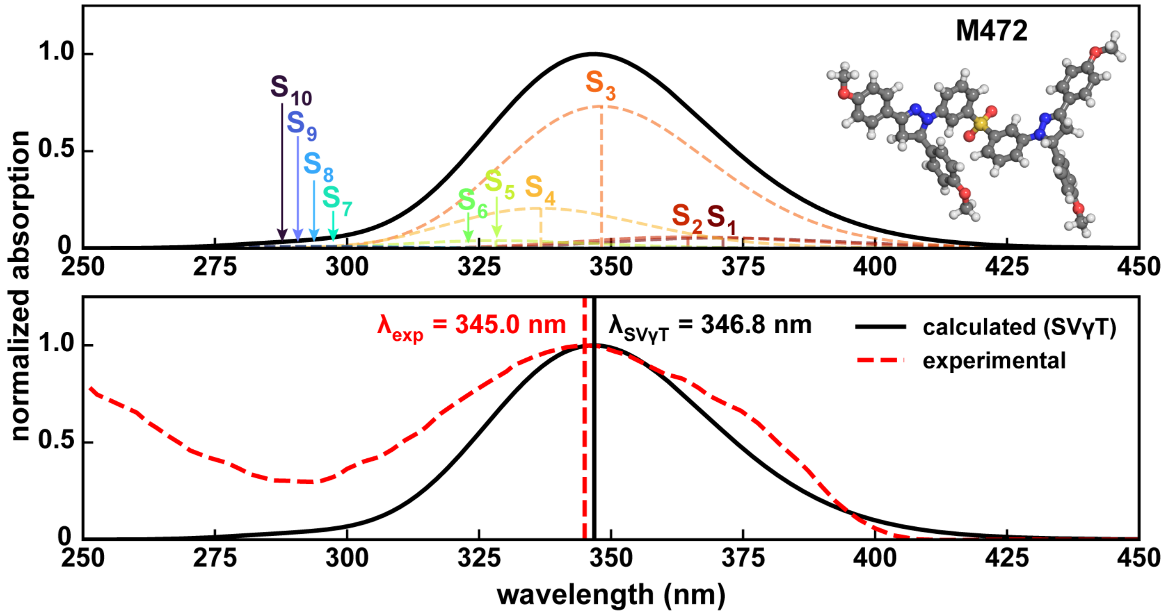

Figure S2. Comparison of the SVγT calculated and experimental spectrum for system M472. (upper) The convolved simulated spectrum (black) and the Gaussian functions correspond to S_1-S_{10}, with arrows and vertical dashed lines to denote the center of each Gaussian. The structure of M472[1] is shown on the right of the panel; (bottom) comparing the convolved (black solid line) and experimental (red dashed line) absorption spectrum, with vertical lines denoting their maxima. The experimental spectrum was extracted from Reference 1 in this document.

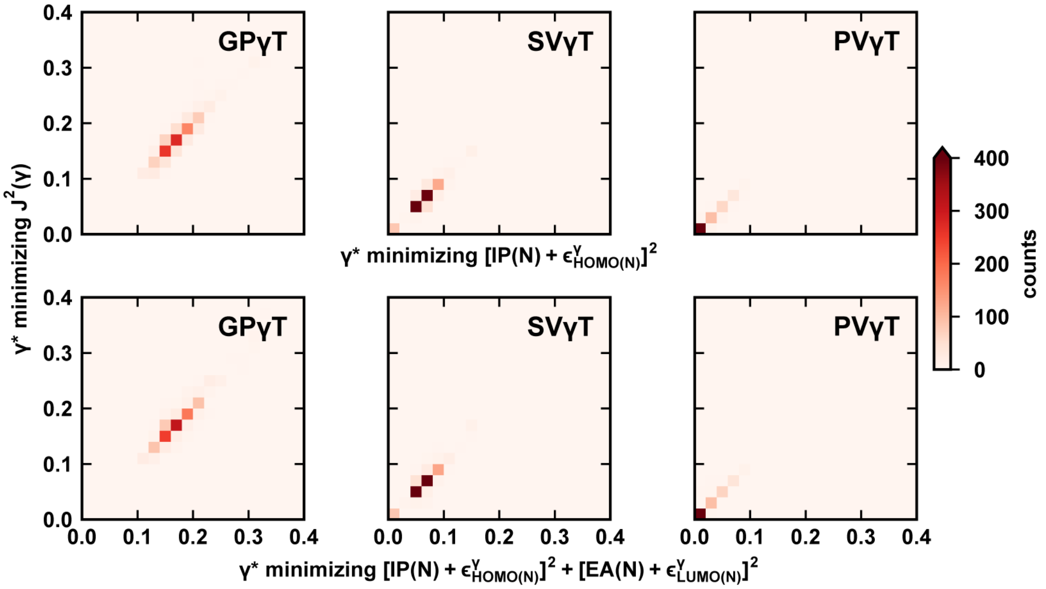

Figure S3. Comparison of optimal γ values obtained using different loss functions in γ tuning procedures. Heatmaps show the distributions of optimal γ values (γ*) for GPγT, PVγT, and SVγT using different loss functions: (i) minimizing only the first term in Eq. (2) [Koopmans' theorem], (ii) minimizing both terms in Eq. (2), and (iii) replacing the second term with $\left[\epsilon^{\gamma}_{\mathrm{LUMO}(N)} + \mathrm{EA}(N;\gamma)\right]^2$. (upper row) comparing γ* obtained by (i) and (ii); (bottom row) comparing γ* obtained by (i) and (iii). The grid spacing used for drawing this figure is 0.02 a_0^{-1}.

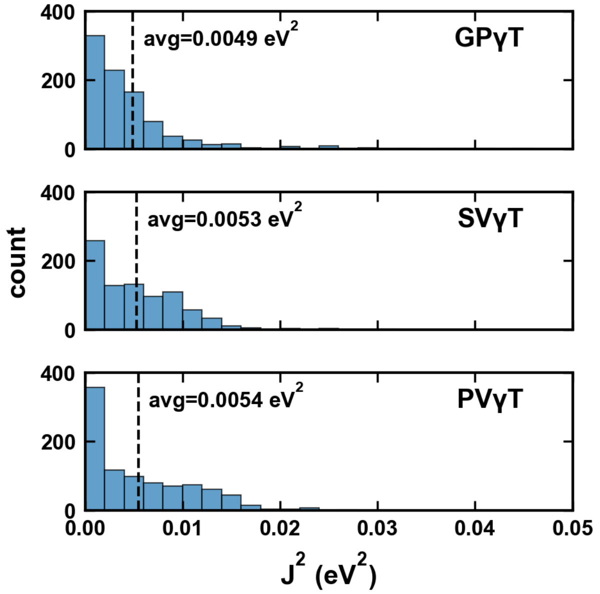

Figure S4. Distribution of the smallest J^2 for three tuning schemes. The average J^2 values are denoted as a vertical dashed line with its value labeled at the right side.

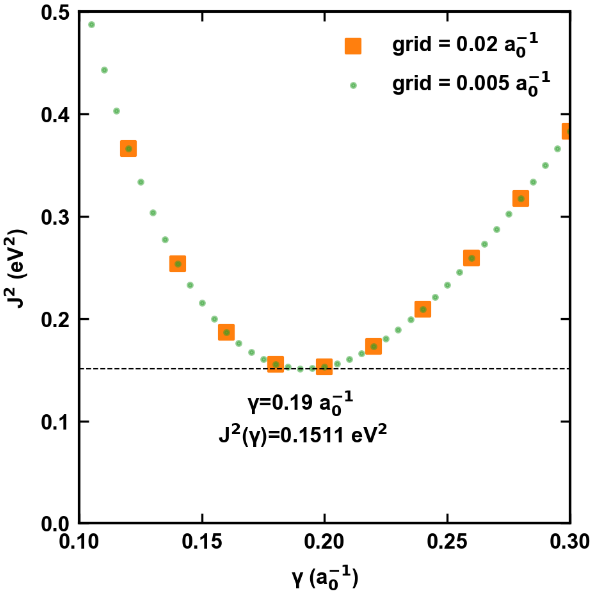

Figure S5. GPγT result for M33. Yellow dots denote the grid search with spacing 0.02 a_0^{-1}, while green dots denote a much finer grid search using spacing 0.005 a_0^{-1}. The horizontal line labels the minimum $J^2(\gamma)$ with $\gamma = 0.19$ a_0^{-1}, while our approach gives $\gamma = 0.20$ a_0^{-1} and $J^2(\gamma) = 0.1536$ eV^2, only 0.002 eV^2 different from the very-fine grid.

Text S1. Proof of the impact of PCM on the HOMO energy and IP for the N+1 anionic state.

The PCM we used here always refers to the conductor-like PCM (C-PCM). In conductor-like PCM, the reaction potential generated by the solute charge distribution is described in terms of an apparent surface charge (ASC) distribution spread over the solute cavity surface. Outside the surface is a dielectric continuum with a dielectric constant □, whereas the cavity containing the solute has a dielectric constant equal to 1. For a given solute molecule, the cavity boundary is usually discretized into M surface segments (tesserae) with an ASCs, $\{q_k^{\infty}\}$, which describes the electric field of the polarized continuum. Give the solute at its neutral (N-electron) state, the corresponding polarization charge, $\mathbf{q}_N$, are determined with a set of linear equations:[2, 3]

$$\mathbf{A}\mathbf{q}_N = -\frac{\varepsilon - 1}{\varepsilon}\mathbf{V}_N. \tag{1}$$

Here, $\mathbf{A} \in \mathbb{R}^{M\times M}$ is the Coulomb interaction between unit polarization charges on two cavity tesserae, which is a positive semi-definite symmetric matrix. $\mathbf{V}_N \in \mathbb{R}^M$ is a vector that represents the solute electrostatic potential at each tessera. Given nuclear charge $\{Q_i\}$ and solution-phase electron density $\rho_{\mathrm{PCM}}^N(\boldsymbol{r})$, the kth element in $\mathbf{V}_N$ can be expressed as:

$$\mathrm{V}_{N,k} = \mathrm{V}_{N,k}^{\mathrm{nuc}} + \mathrm{V}_{N,k}^{\mathrm{el}} = \sum_i \frac{Q_i}{|\boldsymbol{r}_i - \boldsymbol{r}_k|} - \int_a \frac{\rho_{\mathrm{PCM}}^N(\boldsymbol{r})}{|\boldsymbol{r} - \boldsymbol{r}_k|} d\boldsymbol{r}\,, \tag{2}$$

where $\mathrm{V}_{N,k}^{\mathrm{nuc}}$ and $\mathrm{V}_{N,k}^{\mathrm{el}}$ are the solute's nuclear and electronic contributions.

At the equilibrium state, given the electron density $\rho_{\mathrm{PCM}}^N(\boldsymbol{r})$ and the corresponding electrostatic potential $\mathbf{V}_N$, the PCM solvation energy can be expressed as:

$$\Delta E_{\mathrm{PCM}}^{N}[\rho_{\mathrm{PCM}}^{N}(\boldsymbol{r})] = \mathbf{q}_N^{\mathrm{T}}\mathbf{V}_N + \frac{1}{2}\mathbf{q}_N^{\mathrm{T}}\mathbf{A}\mathbf{q}_N + \frac{1}{2}(\frac{1}{\varepsilon-1})\mathbf{q}_N^{\mathrm{T}}\mathbf{A}\mathbf{q}_N \tag{3}$$

$\Delta E_{\mathrm{PCM}}^{N}[\rho_{\mathrm{PCM}}^{N}(\boldsymbol{r})]$ contains the electrostatic interaction between solute and polarization charge (the first term), the self-energy of the polarization charges (the second term), and the work to displace the bound charges to the cavity surface (the third term).

Substituting Eq. (1) into Eq. (3) will give:

$$\Delta E_{\mathrm{PCM}}^{N}[\rho_{\mathrm{PCM}}^{N}(\boldsymbol{r})] = -\frac{1}{2}\frac{\varepsilon-1}{\varepsilon}\mathbf{V}_N^{\mathrm{T}}\mathbf{A}^{-1}\mathbf{V}_N \tag{4}$$

Note that the 1/2 factor exists because the self-energy of polarization charges partially offsets the total solvation energy.

To simplify the problem, we assume that the electron density of the solute does not change significantly upon solvation for both the N, N+1, and N-1 states, i.e., the energy change caused by electron density relaxation can be neglected compared to the solvation energy. Therefore, the electrostatic potential $\mathbf{V}_N$ can be treated as a constant vector, and PCM's impact on the total energy of an N-electron system is given by:

$$\Delta \mathrm{E(N)} \approx -\frac{1}{2}\frac{\varepsilon-1}{\varepsilon}\mathbf{V}_N^{\mathrm{T}}\mathbf{A}^{-1}\mathbf{V}_N \tag{5}$$

And the impact on the ionization potential (IP) of the N+1 cationic state is given by:

$$\Delta \mathrm{IP}\ (N+1) \approx -\frac{1}{2}\frac{\varepsilon-1}{\varepsilon}(\mathbf{V}_N^{\mathrm{T}}\mathbf{A}^{-1}\mathbf{V}_N - \mathbf{V}_{N+1}^{\mathrm{T}}\mathbf{A}^{-1}\mathbf{V}_{N+1}) \tag{6}$$

As we assume PCM does not significantly impact the electron density, the shape of the HOMO of both N, N+1, and N-1 states is also fixed. Hence, the impact of C-PCM on the HOMO of the N+1 anionic state, $\varphi_{\mathrm{HOMO}(N+1)}(\boldsymbol{r})$, can be written as:

$$\Delta\epsilon_{\mathrm{HOMO},(N+1)} = \sum_{\mathrm{k}} q_{N+1,k}\int_a \frac{-\left|\varphi_{\mathrm{HOMO}(N+1)}(\boldsymbol{r})\right|^2}{|\boldsymbol{r}-\boldsymbol{r}_k|}d\boldsymbol{r} \tag{7}$$

Here, $q_{N+1,k}$ is the polarization charge, $\mathbf{q}_N$, on the kth tessera located at $\boldsymbol{r}_k$, and a negative sign is added on the modular square of HOMO because electrons carry a negative charge.

The second assumption we made is the compliance of the one-particle picture, i.e., the ionization of an electron does not affect other electrons' distribution. This means $\rho_{N+1}^{\mathrm{PCM}}(\boldsymbol{r}) - \rho_N^{\mathrm{PCM}}(\boldsymbol{r}) = |\varphi_{HOMO}^{N+1}(\boldsymbol{r})|^2$ holds. Therefore, the $\Delta_{\mathrm{PCM}}\epsilon_{\mathrm{HOMO},(N+1)}$ can be expressed as:

$$\Delta\epsilon_{\mathrm{HOMO},(N+1)} = \sum_{\mathrm{k}} q_{N+1,k}\int_a \frac{-\left(\rho_{\mathrm{PCM}}^{N+1}(\boldsymbol{r}) - \rho_{\mathrm{PCM}}^{N}(\boldsymbol{r})\right)}{|\boldsymbol{r}-\boldsymbol{r}_k|}d\boldsymbol{r}$$

$$= \sum_{\mathrm{k}} q_{N+1,k}\left(\sum_i \frac{Q_i}{|\boldsymbol{r_i}-\boldsymbol{r}_k|} - \int_a \frac{\rho_{\mathrm{PCM}}^{N+1}(\boldsymbol{r})}{|\boldsymbol{r}-\boldsymbol{r}_k|}d\boldsymbol{r}\right) - \sum_{\mathrm{k}} q_{N+1,k}\left(\sum_i \frac{Q_i}{|\boldsymbol{r_i}-\boldsymbol{r}_k|} - \int_a \frac{\rho_{\mathrm{PCM}}^{N}(\boldsymbol{r})}{|\boldsymbol{r}-\boldsymbol{r}_k|}d\boldsymbol{r}\right) \tag{8}$$

Substitute Eq. (2) into Eq. (8)

$$\Delta\epsilon_{\text{HOMO}}(N+1) = \mathbf{q}_{N+1}^{\text{T}}\mathbf{V}_{N+1} - \mathbf{q}_{N+1}^{\text{T}}\mathbf{V}_{N} = -\frac{\varepsilon-1}{\varepsilon}\mathbf{V}_{N+1}^{\text{T}}\mathbf{A}^{-1}\mathbf{V}_{N+1} + \frac{\varepsilon-1}{\varepsilon}\mathbf{V}_{N+1}^{\text{T}}\mathbf{A}^{-1}\mathbf{V}_{N} \quad (9)$$

Therefore, the difference between $-\Delta\epsilon_{\text{HOMO}}(N+1)$ and $\Delta\text{IP}^{\text{CPCM}}(N+1)$ is:

$$-\Delta\epsilon_{\text{HOMO}}(N+1) - \Delta\text{IP}^{\text{CPCM}}(N+1) = \frac{\varepsilon-1}{2\varepsilon}(\mathbf{V}_{N+1} - \mathbf{V}_{N})^{\mathbf{T}}\mathbf{A}^{-1}(\mathbf{V}_{N+1} - \mathbf{V}_{N}) \quad (10)$$

Since the Coulomb matrix, $\mathbf{A}$, is a symmetric positive-definite matrix,[4] its inverse $\mathbf{A}^{-1}$ is also symmetric positive-definite, and the product $\mathbf{x}^{T}\mathbf{A}^{-1}\mathbf{x}$ for an arbitrary vector $\mathbf{x} \neq \mathbf{0}$ is always positive. This means $-\Delta\epsilon_{\text{HOMO},(N+1)} > \Delta\text{IP}\ (N+1)$, explaining the relationship shown in **Figure 4**. Note that this inequality also holds for the ionization potential of the neutral, N-electron state, i.e., $-\Delta\epsilon_{\text{HOMO},(N)} > \Delta\text{IP}^{\text{CPCM}}\ (N)$, where $-\Delta\epsilon_{\text{HOMO},(N)} \approx 0$ but $\Delta\text{IP}\ (N) < 0$.

For an organic molecule with N nuclear charges and N electrons, $\mathbf{V}_{N+1}$ have a much greater magnitude than $\mathbf{V}_N$, **i.e.,** $\mathbf{V}_{N+1} - \mathbf{V}_N \approx \mathbf{V}_{N+1}$. Therefore, the major contribution of Eq. (10) comes from the term including $\mathbf{V}_{N+1}^{\text{T}}\mathbf{A}^{-1}\mathbf{V}_{N+1}$**:**

$$-\Delta\epsilon_{\text{HOMO}}(N+1) - \Delta\text{IP}^{\text{CPCM}}(N+1) \approx \frac{1}{2}\left(\frac{\varepsilon-1}{\varepsilon}\right)\mathbf{V}_{N+1}^{\text{T}}\mathbf{A}^{-1}\mathbf{V}_{N+1} = \frac{1}{2}\left(\frac{\varepsilon}{\varepsilon-1}\right)\mathbf{q}_{N}^{\text{T}}\mathbf{A}\mathbf{q}_{N} \quad (11)$$

And Eq. (11) can be rewritten as:

$$\frac{1}{2}\left(\frac{\varepsilon}{\varepsilon-1}\right)\mathbf{q}_{N}^{\text{T}}\mathbf{A}\mathbf{q}_{N} = \frac{1}{2}\mathbf{q}_{N}^{\text{T}}\mathbf{A}\mathbf{q}_{N} + \frac{1}{2}\left(\frac{1}{\varepsilon-1}\right)\mathbf{q}_{N}^{\text{T}}\mathbf{A}\mathbf{q}_{N} \quad (12)$$

This term equals the self-energy of the polarization charge plus the work required to induce it, as discussed in Eq. (3). For polar solvents with $\varepsilon \gg 1$, the major difference between $-\Delta\epsilon_{\text{HOMO}}(N+1)$ and $\Delta\text{IP}^{\text{CPCM}}(N+1)$ originates from self-energy.

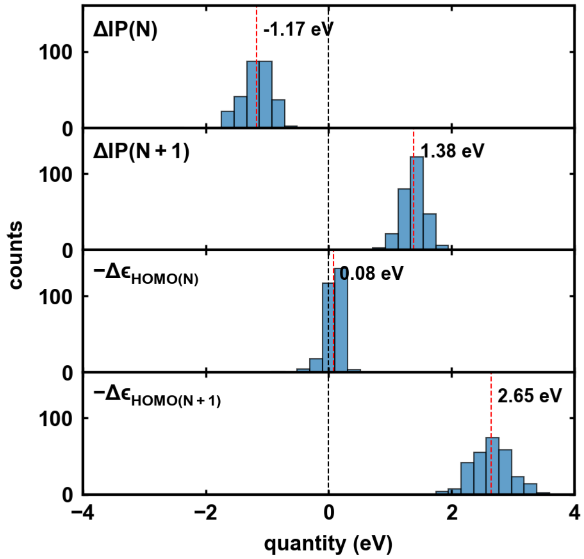

Figure S6. Change in the HOMO energies and IP after applying PCM to the DCM solvent group. The quantities tested here are $\text{IP}(N;\ \gamma)$, $\text{IP}(N+1;\ \gamma)$, $-\epsilon_{\text{HOMO}(N)}^{\gamma}$, and $-\epsilon_{\text{HOMO}(N+1)}^{\gamma}$, with Δ added before all mathematical symbols to indicate they are the changes after applying PCM with ε=8.93. All quantities are evaluated using a fixed γ=0.20 a_0^{-1}. The red dashed vertical

line labels the average change for each quantity over 251 molecules, while the black vertical dashed line marks the position of zero.

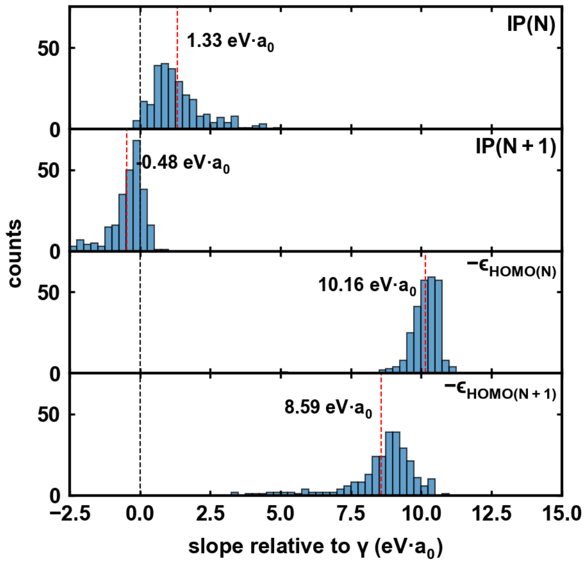

Figure S7. Slope of HOMO energies and IP relative to γ for the DCM solvent group. All quantities are evaluated using a fixed ε=8.93. γ is uniformly sampled from 0.0-0.2a_0^{-1} with an interval of 0.02a_0^{-1}. The red dashed vertical line labels the average slopes for each quantity over 251 molecules, while the black dashed line labels the zero position.

Text S2. Assessing the impact of solvent polarity on optimal γ under SVγT.

When using non-equilibrium solvation, the polarization charge for both N and N+1 electron states can be divided into fast and slow parts; the slow part scales with $(\varepsilon_\infty - 1)/\varepsilon_\infty$, therefore,

$$\mathbf{q}_N = \mathbf{q}_N^{\mathbf{fast}} + \mathbf{q}_N^{\mathbf{slow}} = \frac{\varepsilon_\infty - 1}{\varepsilon_\infty}\mathbf{q}_N^\infty + (\frac{\varepsilon - 1}{\varepsilon} - \frac{\varepsilon_\infty - 1}{\varepsilon_\infty})\mathbf{q}_N^\infty \tag{13}$$

Here, $\mathbf{q}_N^\infty$ denotes the polarization charge generated by a medium with infinite dielectric constant.

$$\mathbf{q}_N^\infty = -\mathbf{A}^{-1}\mathbf{V}_N \tag{14}$$

During the Ionization process, $\mathbf{q}_N^{\mathbf{slow}}$ remains fixed, while only $\mathbf{q}_N^{\mathbf{fast}}$ respond to the ionization process and become $\mathbf{q}_{N+1}^{\mathbf{fast}}$. Retain the assumption in Text S1, for an organic molecule with N nuclear charges and N electrons, $\mathbf{V}_{N+1}$ have a much greater magnitude than $\mathbf{V}_N$, which means $\mathbf{q}_{N+1}^\infty$ also has a much greater magnitude than $\mathbf{q}_N^\infty$.

For most organic solvents, such as the 9 solvents used in our case, their fast dielectric constant ε_∞ ranges from 1.76 (methanol) to 2.24 (toluene), which means the perfector $\frac{\varepsilon_\infty - 1}{\varepsilon_\infty}$ of $\mathbf{q}_N^{\mathbf{fast}}$ ranges from 0.43 to 0.55. For the most polar solvent DMSO, its static dielectric constant is 46.7, so the perfector of the $\mathbf{q}_N^{\mathbf{slow}}$, $\frac{\varepsilon - 1}{\varepsilon} - \frac{\varepsilon_\infty - 1}{\varepsilon_\infty}$, is approximately 0.435, and for the typical non-polar solvent like cyclohexane, $\mathbf{q}_N^{\mathbf{slow}}$ is nearly zero as the solvent molecule does not bear any static

dipole moments. As the perfector of $\mathbf{q}_N^{\mathbf{slow}}$ and $\mathbf{q}_{N+1}^{\mathbf{fast}}$ is always under the same magnitude, while $|\mathbf{q}_N^{\infty}| \ll |\mathbf{q}_{N+1}^{\infty}|$, we can conclude that $|\mathbf{q}_N^{\mathbf{slow}}| \ll |\mathbf{q}_{N+1}^{\mathbf{fast}}|$, for all of our solvents.

This conclusion means that, in the case of non-equilibrium solvation, the effect of the PCM on $\epsilon_{\mathrm{HOMO},(N)}$, $\epsilon_{\mathrm{HOMO},(N+1)}$, IP (N), and IP $(N+1)$ is very close to that of a nonpolar solvent with ε = ε_{∞}. Since the ε_{∞} of the solvents we used fluctuated only within a small range; the effect of all solvents on optimal γ values is similar.

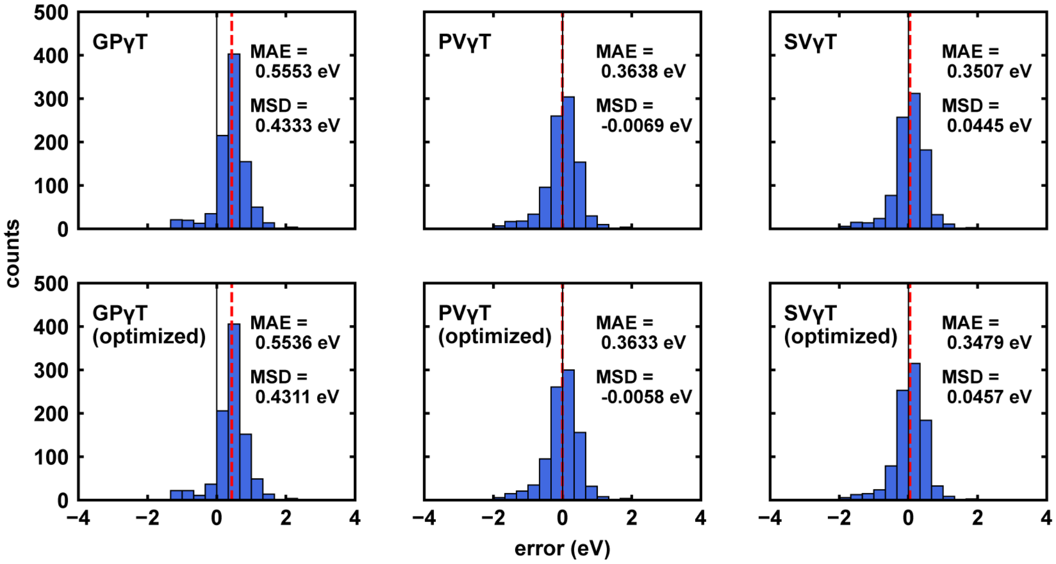

Figure S8. Impact of geometry relaxation on the performance of different schemes. (upper row). The distribution of prediction errors for each γ-tuning scheme. The MSD is indicated by a dashed red line, while a vertical reference line marks the ideal predictive performance (zero error); (bottom row), the prediction error for each scheme after optimizing the geometry using their optimal γ values. The MAE and MSD are rounded to 4 decimal places for better comparison.

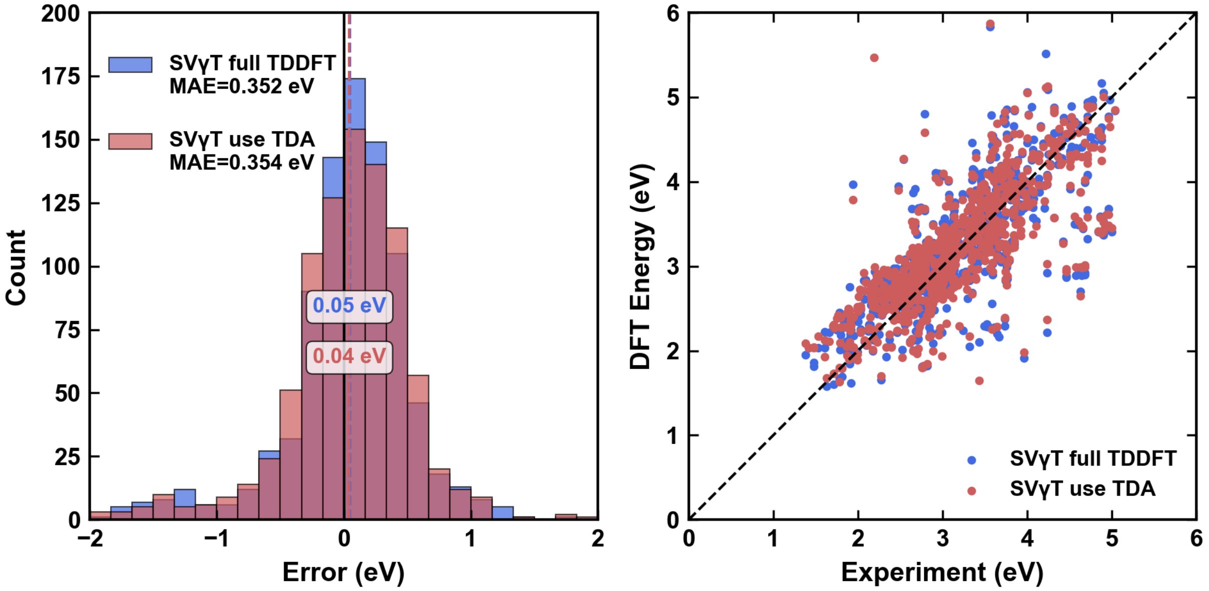

Figure S9. Impact of TDA on SVγT. (left) The distribution of prediction errors of SVγT when using full TDDFT and with TDA enabled. The MSDs are denoted by blue (full TDDFT) and red (TDA) text and vertical dashed lines. (right) Parity plots compare experimental results with SVγT using full TDDFT (blue) and TDA (red). The ideal agreement between experimental and predicted values is represented by diagonal dashed lines, denoting the parity line.

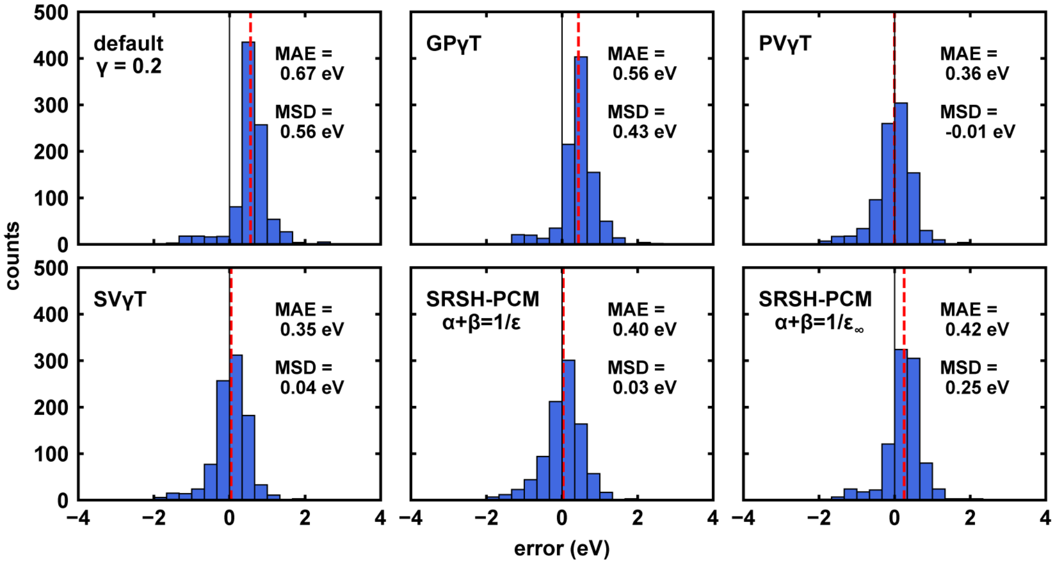

Figure S10. Comparison of the two variants of SRSH-PCM with other γ-tuning schemes. The MSD is indicated by a dashed red line, while a vertical reference line marks the ideal predictive performance (zero error).

Text S3. Procedure of tuning the short-range exact exchange fraction (α) and the range-separation parameter (γ) together.

To evaluate the effect of simultaneously tuning the short-range exact exchange fraction (α) and the range-separation parameter (γ), we performed a two-dimensional parameter scan following the general protocol of sol-med-OT with modifications for our study.[5] Specifically, 21 molecules solvated in dichloromethane (DCM, $\varepsilon = 9.02$, $\varepsilon_{¥} = 2.03$) were randomly selected from the DCM group containing 240 solvents. The standard procedure is illustrated schematically in Figure S11. In step 1 (left branch of the workflow), α was varied from 0.00 to 0.45 in increments of 0.05. For each α, γ was scanned from 0.00 to 0.45 with a coarse step of 0.05 to evaluate the target function J^2 in the gas phase. For each α, a fine grid (step size 0.01) was used to re-scan γ around the minimum region identified in the previous step, yielding the locally optimal value γ*. After obtaining (α, γ*) in step 1, we turn on the non-equilibrium PCM; the complementary fraction was set to $\beta = 1/\varepsilon_{¥} - \alpha$, and J^2 was recalculated using the non-equilibrium PCM model. Among all candidate pairs (α, γ*), the combination that minimized J^2 was selected as the final optimal parameter set (α*, γ*). For comparison, in the SVγT scheme, the PCM model is included from the beginning of the two-parameter scan.

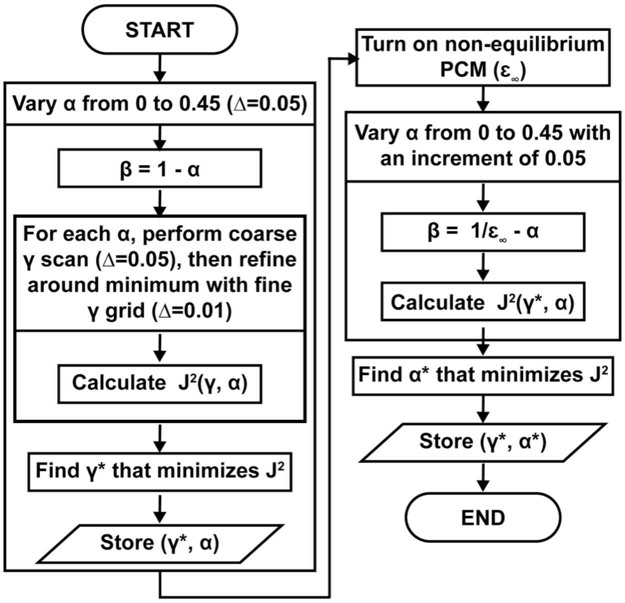

Figure S11. Workflow for two-parameter tuning of (α, γ) in the sol-med-OT framework. For each system, α varies from 0.00 to 0.45 in increments of 0.05. At each α, a coarse scan of γ from 0.00 to 0.45 is performed, followed by a fine grid refinement (step 0.01) near the minimum to identify the locally optimal γ*. In the non-equilibrium PCM environment, the complementary fraction is set to $\beta = 1/\varepsilon_{¥} - \alpha$, and J^2 is recomputed. Among all candidate pairs (α, γ*), the combination that minimizes J^2 is selected as the final optimal parameter set (α*, γ*). This workflow allows both short-range exact exchange and range-separation to be tuned consistently under solvent screening.

Table S1. Comparison of tuned parameters (α, γ) and excitation energies for 21 representative molecules in DCM (ε = 9.02, ε∞ = 2.03). Columns list the short-range exact exchange fraction (α) and optimal range-separation parameter (γ*) obtained from solvent γ tuning (SVγT), together with the corresponding optimal parameters (α*, γ*) from the sol-med-OT workflow. Calculated excitation energies are reported for SVγT (α+β=1), SVγT (α+β=1/ε∞), sol-med-OT (α+β=1), and sol-med-OT (α+β=1/ε∞), alongside experimental reference values. Statistical measures include the mean absolute error (MAE) and mean signed deviation (MSD) calculated for the excitation energy with respect to experiment. Bold numbers indicate the method with the smallest MAE and MSD, and "N.A." stands for "Not Applicable".

Molecule	a (SVγT)	γ* (SVγT) (a_0^{-1})	a* (sol-med-OT)	γ* (sol-med-OT) (a_0^{-1})	SVγT (a+b=1) (eV)	SVγT (a+b=1/ε∞) (eV)	sol-med-OT (a+b=1) (eV)	sol-med-OT (a+b=1/ε∞) (eV)	Exp. (eV)
M319	0.2	0.06	0.35	0.14	2.63	2.62	3.10	2.96	2.43
M320	0.2	0.07	0.4	0.15	4.07	4.04	4.47	4.37	3.69
M358	0.2	0.05	0.3	0.12	2.98	2.97	3.14	3.08	2.79
M359	0.2	0.05	0.3	0.12	2.91	2.89	3.12	3.04	2.82
M366	0.2	0.05	0.3	0.13	2.97	2.94	3.51	3.29	3.27
M368	0.2	0.05	0.3	0.12	3	2.99	3.21	3.14	2.9
M393	0.2	0.06	0.3	0.14	3.62	3.59	3.96	3.83	3.25
M428	0.2	0.06	0.35	0.14	2.65	2.62	3.1	2.95	2.43
M436	0.2	0.05	0.3	0.13	3.05	3.03	3.29	3.2	2.9
M478	0.2	0.06	0.4	0.13	4.29	4.24	4.86	4.69	4.4
M501	0.2	0.06	0.35	0.13	2.56	2.44	3.35	2.98	2.87
M503	0.2	0.05	0.3	0.14	2.42	2.34	3.01	2.69	2.58
M511	0.2	0.06	0.35	0.14	3.25	3.17	3.85	3.59	3.25
M529	0.2	0.06	0.35	0.14	3.35	3.32	3.74	3.62	3.35
M549	0.2	0.06	0.35	0.15	4.38	4.36	4.72	4.6	4.12
M555	0.2	0.07	0.4	0.14	3.36	3.28	3.97	3.76	3.3
M559	0.2	0.06	0.35	0.14	3.22	3.2	3.61	3.49	2.88
M565	0.2	0.05	0.3	0.13	3	2.97	3.31	3.18	2.85
M566	0.2	0.05	0.35	0.13	2.97	2.94	3.27	3.18	3.27
M580	0.2	0.05	0.3	0.12	2.71	2.68	3.01	2.88	2.7
M616	0.2	0.05	0.35	0.12	3.38	3.35	3.85	3.7	3.54
MAE	N.A.	N.A.	N.A.	N.A.	**0.18**	0.19	0.47	0.32	0.00
MSD	N.A.	N.A.	N.A.	N.A.	0.06	**0.02**	0.47	0.31	0.00

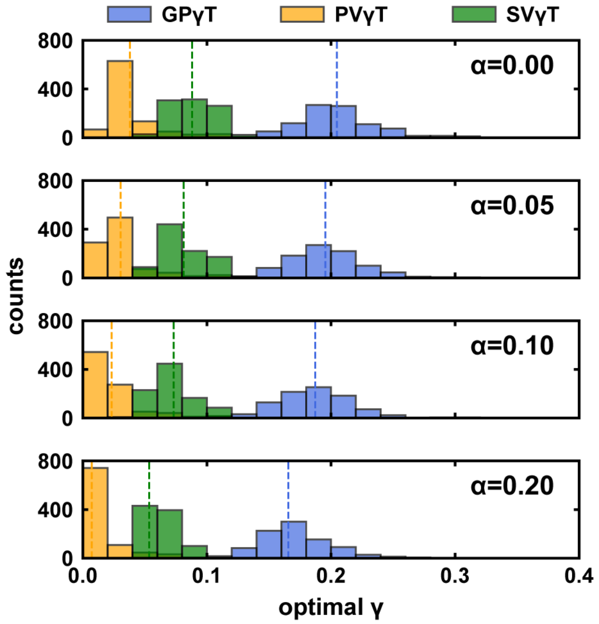

Figure S12. Distribution of optimal γ values for different γ-tuning schemes with different α. The mean γ-values for each scheme are shown in vertical dashed lines.

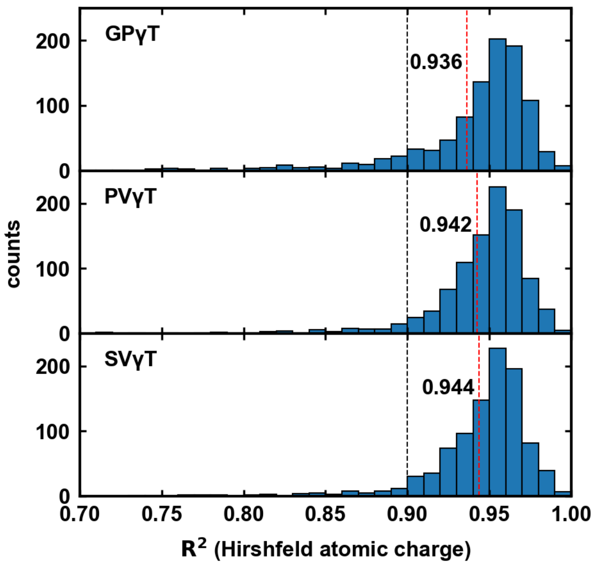

Figure S13. Result for one particle picture test of different γ-tuning schemes. The average R^2 for atomic Hirshfeld charges over 251 molecules is labeled by the red dashed vertical line. The black vertical dashed line indicates the threshold of R^2=0.90.

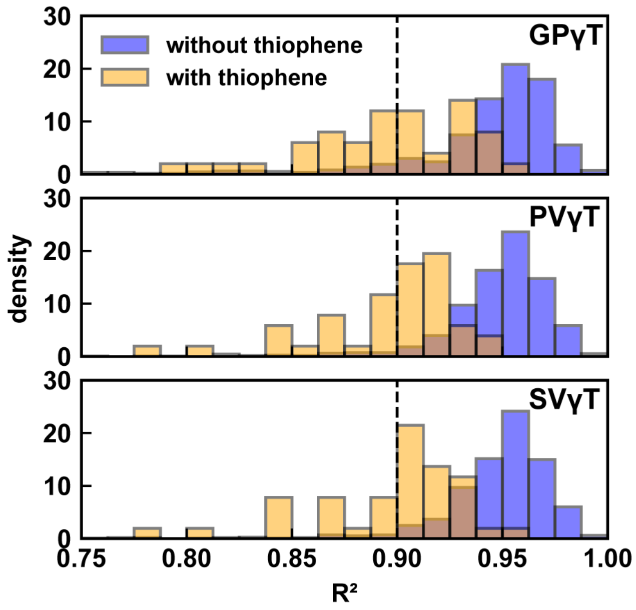

Figure S14. Impact of thiophene on one-particle picture compliance. Density distribution of R^2 values between the Hirshfeld population for GPγT, PVγT, and SVγT, comparing molecules without (blue) and with (orange) thiophene rings. The black vertical dashed line indicates the threshold of R^2=0.90. There are a total 101 out of 937 solutes containing rings of thiophene.

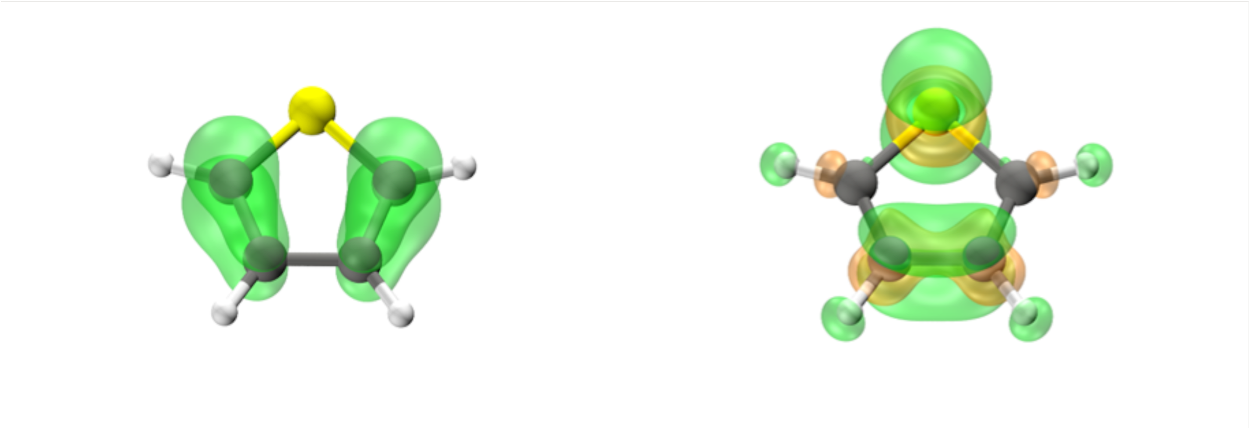

Figure S15. Comparison of thiophene's HOMO density and electron density change after ionization. (left) $\left|\phi^N_{\mathrm{HOMO}}(\vec{r})\right|^2$; (right) $\Delta\rho(\vec{r})=\rho^N(\vec{r})-\rho^{N-1}(\vec{r})$. Green and orange indicate positive and negative values in real-space density. This plot is generated by VMD using grid spacing 0.05 a.u. and isosurfaces value ± 0.005 a.u. Atom color coding: white-hydrogen, gray-carbon, yellow-sulfur.

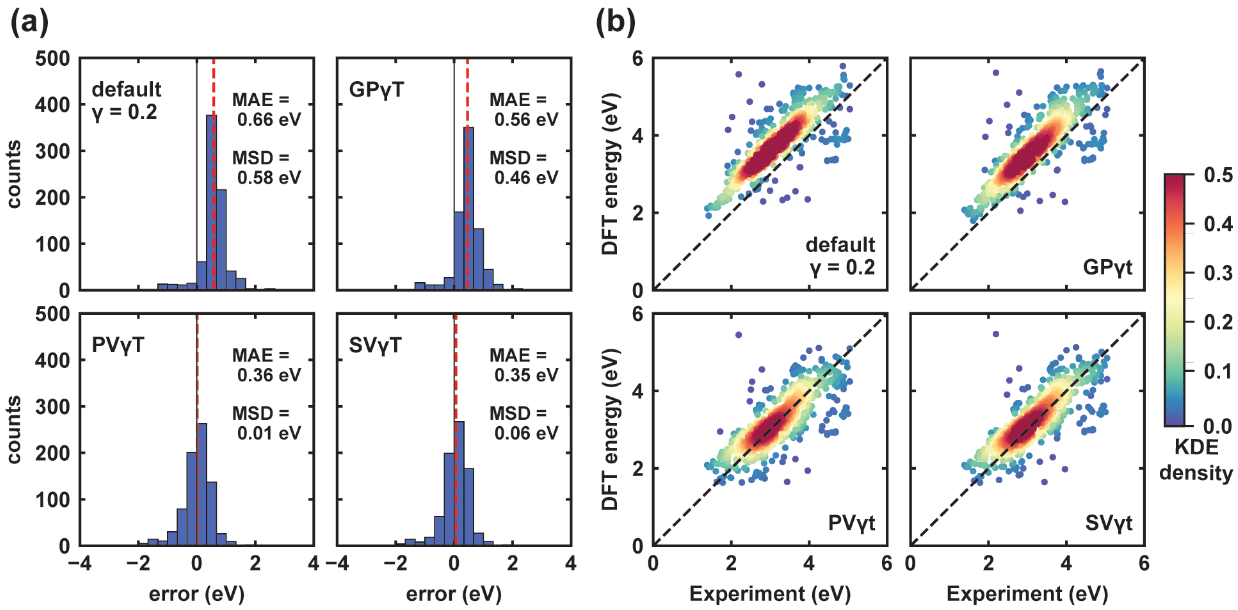

Figure S16. Performance of different γ-tuning schemes only for entries in compliance with the one-particle picture. (a) Histogram plots illustrate the distribution of prediction errors for each γ-tuning scheme. The MSD is denoted by a dashed red line, while a vertical reference line marks the ideal predictive performance (zero error). (b) Parity plots compare experimental results with predicted values across different γ-tuning schemes. Data point densities are color-coded based on Kernel Density Estimation (KDE) values, as shown in the right color bar. The ideal agreement between experimental and predicted values is represented by diagonal dashed lines, denoting the parity line.

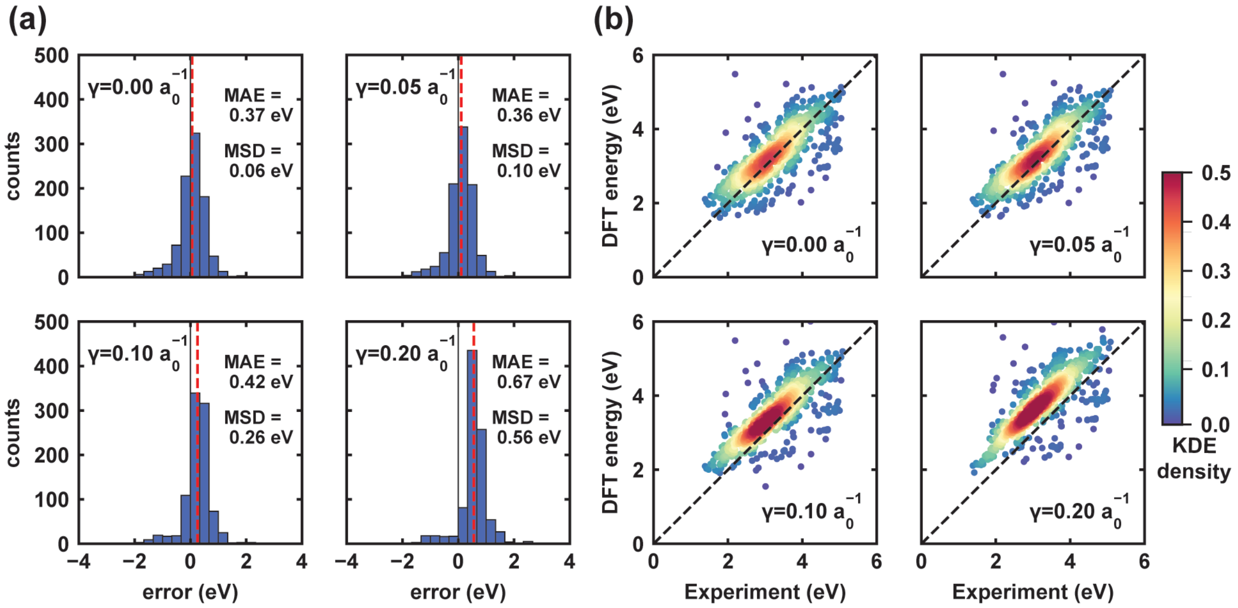

Figure S17. Performance of using fixed γ values. (a) Histogram plots illustrate the distribution of prediction errors for each γ-tuning scheme. The MSD is denoted by a dashed red line, while a

vertical reference line marks the ideal predictive performance (zero error). (b) Parity plots compare experimental results with predicted values across different γ-tuning schemes. Data point densities are color-coded based on Kernel Density Estimation (KDE) values, as shown in the right color bar. The ideal agreement between experimental and predicted values is represented by diagonal dashed lines, denoting the parity line.

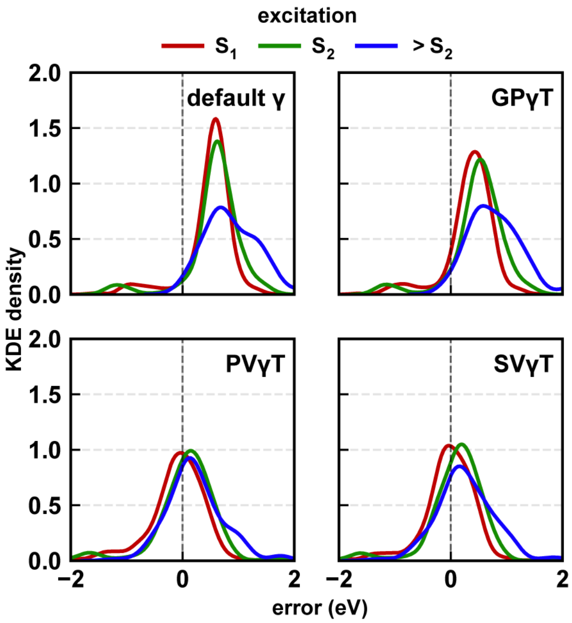

Figure S18. KDE plot of error distribution classified by the excited state associated with the simulated absorption peak.

Text S4. Procedure for generating the explicit solvent cluster for M1074.

All Molecular dynamics simulation were carried out using the AMBER suite.[6] The initial structure for M1074 is taken from the DFT optimized structure, with its parameterization done by the antechamber suite in AmberTools.[7] The atomic charges are assigned by the AM1-BCC method,[8, 9] with the bond, angle, and dihedral parameter obtained from the general amber forcefield (GAFF).[10] The solvation was done by tleap using AMBER's prebuilt methanol solvent box, resulting in a 30x30x30 Å solvent box.

Molecular dynamics simulations were carried out using the sander engine. The solute's conformation was frozen during all steps of simulation, to exclude the impact of solute conformation fluctuation on the excitation energy. Starting from a 1000 steps energy minimization until the maximum force smaller than 0.01 kcal / (mol·Å), followed by 100 ps heat up to increase the temperature to 300 K. After that, a 1 ns NPT simulation was carried out with the barostat enabled.[11] All simulations used a timestep 1 fs. Only the last snapshot of the trajectory will be used for generating the explicit solvation configuration.

The explicit solvation configuration was extracted using AutoSolvate,[12] where the cutoff of the closest distance between the solute and solvent is set to 2.5 Å, only including solvent molecules that forms a medium to strong hydrogen bonding with the solvent.

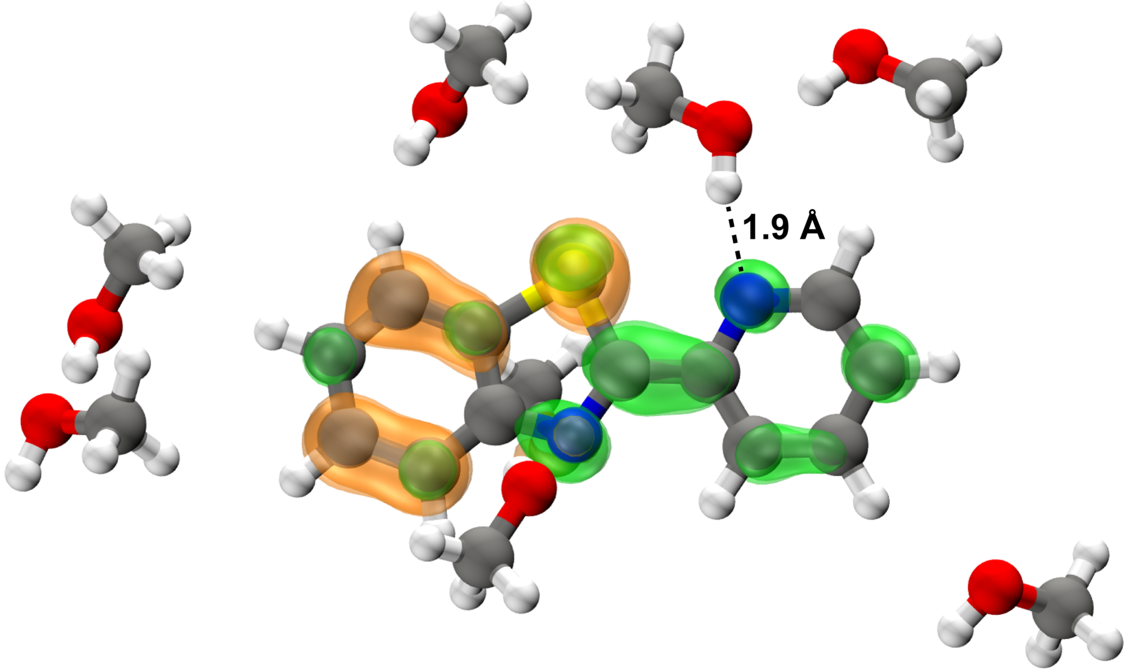

Figure S19. HOMO and LUMO density of solvated M1074. Green and orange indicate HOMO and LUMO density, respectively. A black dashed line draws a hydrogen bond with length 1.9 Å formed between a methanol and the pyridine nitrogen of M1074. This plot is generated by VMD using grid-spacing of 0.05 a.u. and isosurfaces with a value of ± 0.005 a.u. Atom color coding: white-hydrogen, gray-carbon, blue-nitrogen, and red-oxygen.

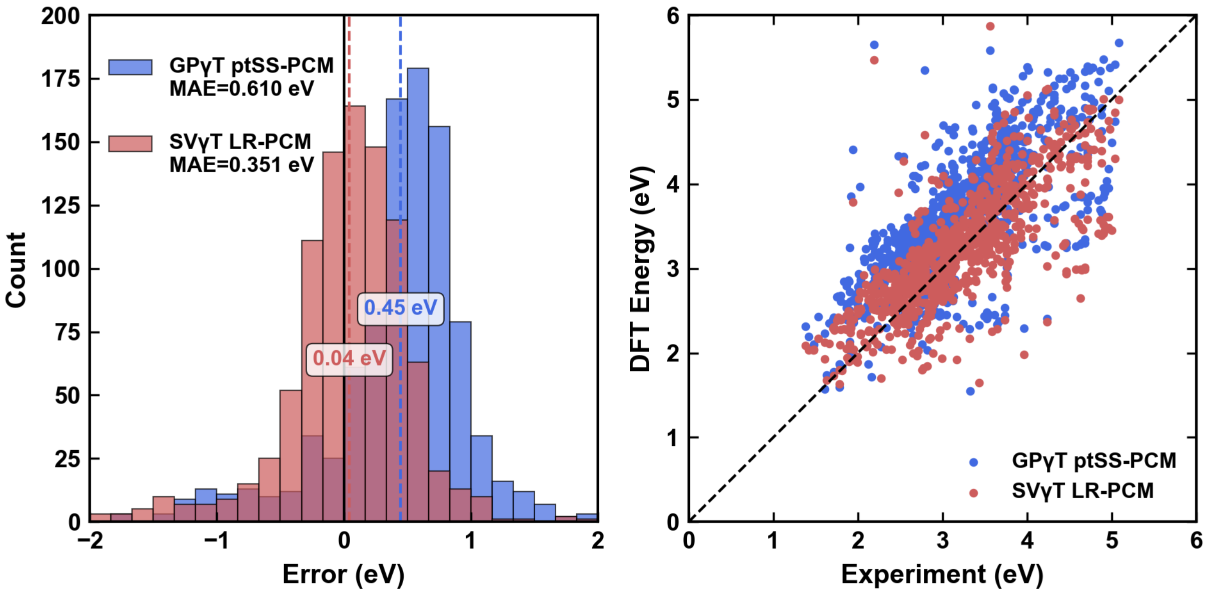

Figure S20. Comparison of GPγT using ptSS-PCM and SVγT. (left) The distribution of prediction errors of GPγT + ptSS-PCM and SVγT + neq-LR-PCM. The MSDs are denoted by blue (full TDDFT) and red (TDA) text and vertical dashed lines. (right) Parity plots compare

experimental results with GPγT + ptSS-PCM (blue) and SVγT + neq-LR-PCM (red). The ideal agreement between experimental and predicted values is represented by diagonal dashed lines, denoting the parity line.

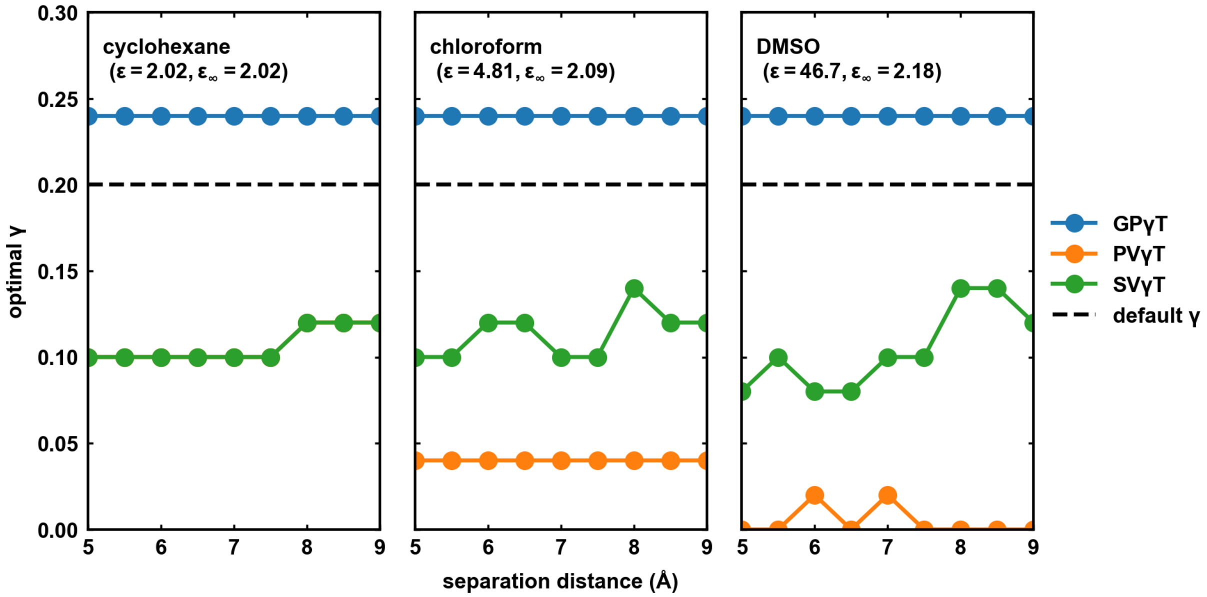

Figure S21. Optimal γ for the ETH-TFE dimers with respect to their separation distance in different solvents. Note, since the ε and ε_∞ for cyclohexane are nearly identical, PVγT and SVγT give the same optimal γ for all ETH-TFE dimers in cyclohexane.

References

(1) Gong, Z.-L.; Zheng, L.-W.; Zhao, B.-X. Synthesis, X-ray crystal structure and optical properties research of novel diphenyl sulfone-based bis-pyrazoline derivatives. *Journal of Luminescence* **2012**, *132* (2), 318-324. DOI: 10.1016/j.jlumin.2011.08.037.

(2) Lange, A. W.; Herbert, J. M. A smooth, nonsingular, and faithful discretization scheme for polarizable continuum models: The switching/Gaussian approach. *The Journal of Chemical Physics* **2010**, *133* (24), 244111. DOI: 10.1063/1.3511297 (acccessed 2023-05-28 04:22:23).Silverchair.

(3) Mennucci, B. Polarizable continuum model. *Wiley Interdisciplinary Reviews: Computational Molecular Science* **2012**, *2* (3), 386-404.

(4) Scalmani, G.; Frisch, M. J. Continuous surface charge polarizable continuum models of solvation. I. General formalism. *The Journal of Chemical Physics* **2010**, *132* (11), 114110. DOI: 10.1063/1.3359469.

(5) Joo, B.; Han, H.; Kim, E.-G. Solvation-Mediated Tuning of the Range-Separated Hybrid Functional: Self-Sufficiency through Screened Exchange. *Journal of Chemical Theory and Computation* **2018**, *14* (6), 2823-2828. DOI: 10.1021/acs.jctc.8b00049 (acccessed 2024-12-13 20:28:10).ACS Publications.

(6) Case, D. A.; Aktulga, H. M.; Belfon, K.; Ben-Shalom, I.; Brozell, S. R.; Cerutti, D. S.; Cheatham III, T. E.; Cruzeiro, V. W. D.; Darden, T. A.; Duke, R. E. *Amber 2021*; University of California, San Francisco, 2021.
(7) Case, D. A.; Aktulga, H. M.; Belfon, K.; Cerutti, D. S.; Cisneros, G. A.; Cruzeiro, V. W. D.; Forouzesh, N.; Giese, T. J.; Götz, A. W.; Gohlke, H. AmberTools. *Journal of chemical information and modeling* **2023**, *63* (20), 6183-6191.
(8) Besler, B. H.; Merz Jr., K. M.; Kollman, P. A. Atomic charges derived from semiempirical methods. *Journal of Computational Chemistry* **1990**, *11* (4), 431-439. DOI: 10.1002/jcc.540110404 (acccessed 2022-10-09 16:34:41).Wiley Online Library.
(9) Jakalian, A.; Jack, D. B.; Bayly, C. I. Fast, efficient generation of high-quality atomic charges. AM1-BCC model: II. Parameterization and validation. *Journal of Computational Chemistry* **2002**, *23* (16), 1623-1641. DOI: https://doi.org/10.1002/jcc.10128 (acccessed 2024/04/02).
(10) Wang, J.; Wolf, R. M.; Caldwell, J. W.; Kollman, P. A.; Case, D. A. Development and testing of a general amber force field. *Journal of Computational Chemistry* **2004**, *25* (9), 1157-1174. DOI: 10.1002/jcc.20035 (acccessed 2023-05-28 03:47:29).Wiley Online Library.
(11) Berendsen, H. J.; Postma, J. v.; Van Gunsteren, W. F.; DiNola, A.; Haak, J. R. Molecular dynamics with coupling to an external bath. *The Journal of chemical physics* **1984**, *81* (8), 3684-3690.
(12) Hruska, E.; Gale, A.; Huang, X.; Liu, F. AutoSolvate: A toolkit for automating quantum chemistry design and discovery of solvated molecules. *The Journal of Chemical Physics* **2022**, *156* (12), 124801. DOI: 10.1063/5.0084833 (acccessed 2023-01-19 03:15:53).DOI.org (Crossref).
